# New α-glucosidase inhibitors from marine algae-derived *Streptomyces* sp. OUCMDZ-3434

**DOI:** 10.1038/srep20004

**Published:** 2016-01-29

**Authors:** Zhengbo Chen, Jiejie Hao, Liping Wang, Yi Wang, Fandong Kong, Weiming Zhu

**Affiliations:** 1 Key Laboratory of Marine Drugs, Ministry of Education of China, School of Medicine and Pharmacy, Ocean University of China, Qingdao 266003, China; 2 Key Laboratory of Chemistry for Natural Products of Guizhou Province and Chinese Academy of Sciences, Guiyang 550002, China

## Abstract

Wailupemycins H (**1**) and I (**2**) with a new skeleton coupled two 6-(2-phenylnaphthalene-1-yl)pyrane-2-one nuclei to a –CH_2_– linkage were identified from the culture of *Streptomyces* sp. OUCMDZ-3434 associated with the marine algae, *Enteromorpha prolifera*. Compounds **1** and **2** are two new α-glucosidase inhibitors with the K_i_/IC_50_ values of 16.8/19.7 and 6.0/8.3 *μ*M, respectively. In addition, the absolute configurations of wailupemycins D (**3**) and E (**4**) are also resolved in this paper for the first time.

Diabetes mellitus is a clinical syndrome caused by genetic factors and environmental factors and characterized by high levels of blood glucose. For the past few years, the incidence of diabetes increased rapidly, and the diabetes treatment has become a global health problem^1^. Over 85% diabetics suffer from Type-II diabetes mellitus (DM)^2^. DM is a chronic disease with clinical manifestation of hyperglycemia, due to the ineffective of insulin that controls the level of blood glucose. α-Glucosidase inhibitors, such as acarbose, voglibose and miglitol, could retard the uptake of dietary carbohydrates and have achieved significant therapeutic effect in clinical application^3^. However, most of these products have side effects such as flatulence, nausea, vomiting and diarrhea^4^. Therefore, more secure and efficient inhibitors are the urgent demand for the treatment of diabetes.

Marine is believed to be a natural treasure house for medicine, and natural products of marine microbial origin play an increasingly important role in drug diescovery^5^. In our ongoing search for bioactive natural products with new structures from marine microorganisms^6^,^7^,^8^, the EtOAc extract of the fermentation broth of *Streptomyces* sp. OUCMDZ-3434 associated with the marine green algae, *Enteromorpha prolifera*, exhibited significant α-glucosidase inhibitory activity at 50 *μ*g/mL. Chemical study lead to the identification of two new epimeric polyketides that we named wailupemycins H (**1**) and I (**2**) with an unusual carbon skeleton coupled two 6-(2-phenylnaphthalene-1-yl)pyrane-2-one nuclei into a methylene linkage, along with the known wailupemycins D (**3**)^9^, E (**4**),^10^ and G (**5**)^10^ (Fig. 1).

## Results

The constitutions and the relative configurations of the known compounds **3** and **4** were identical to those of wailupemycins D^9^ and E^10^ by comparing their NMR data (Table S1) and NOESY spectra, respectively (Figures S26 and S30). However, their absolute configurations have not been resolved yet in the literature. So we determined their absolute configurations by CD and ECD calculations of **3**, *ent*-**3** (Fig. 2), **4**, and *ent*-**4** (Fig. 3). The results showed that the CD spectra of **3** and **4** shared the almost identical Cotton effects with the calculated ECD, but opposite to the calculated ECD of *ent-***3** and *ent*-**4**, respectively. The absolute configurations of wailupemycins D (**3**) and E (**4**) were thus elucidated as (6*S*, 15*R*)- and (6*R*, 15*R*)-, respectively. The 15*R*- configuration of **3** was confirmed by Horeau’s method^11^,^12^,^13^. After reaction with (±)2-phenylbutyric chloride, the recovered 2-phenylbutyric acid (2-PHA) showed positive specific rotation, indicating the *S*-enantiomeric excess of 2-PHA. To analyze the results, the quantum calculation of molecular energies on the products of **3** with 2*R*- and 2*S*- PHA (**3a** and **3b**) was carried out by the time-dependent density functional theory (TD-DFT) method at the B3LYP/6-31G(d) level^8^. The calculation showed that the molecular energy of **3a** is lower than **3b**. (Table S2, Supporting Information), indicating the higher reaction rate of **3** with 2*R*-PHA agreed with the Horeau’s reaction result. Thus, the 15*R*- configuration of **3** was further supported.

**Figure 1:**
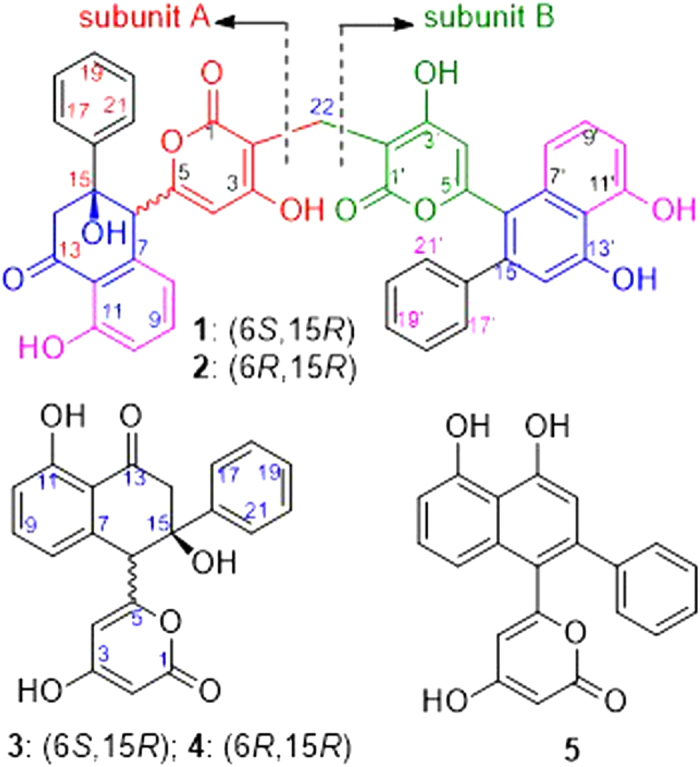
Structures of compounds 1−5.

**Figure 2:**
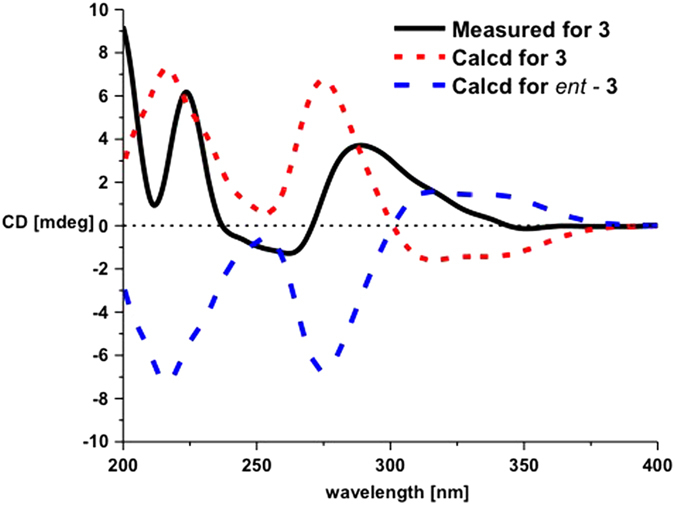
CD and ECD curves of 3 and *ent*-3.

**Figure 3:**
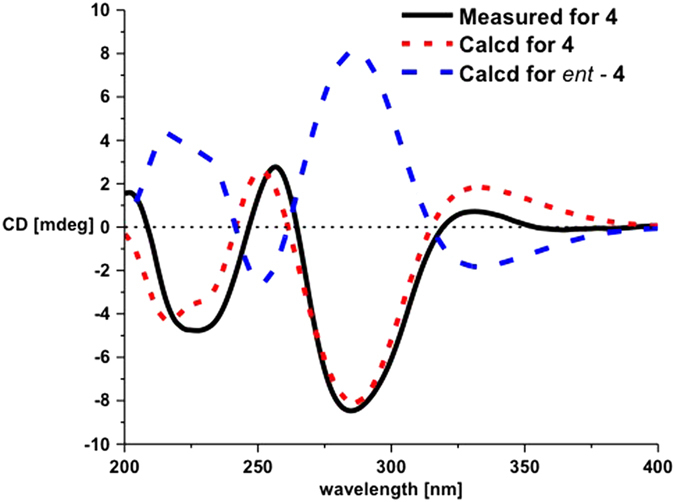
CD and ECD curves of 4 and *ent-*4.

**Figure 4:**
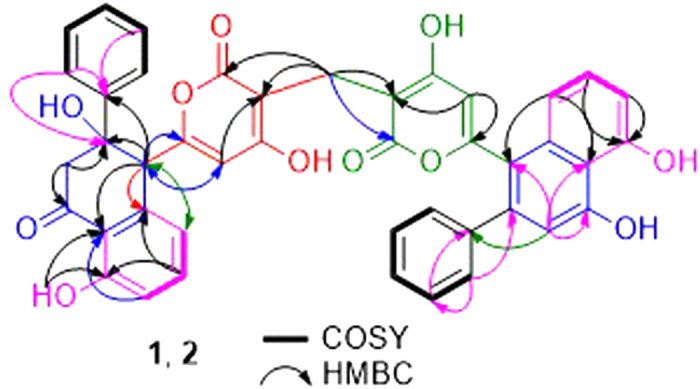
Key HMBC, ^1^H-^1^H COSY correlations of 1 and 2.

**Figure 5:**
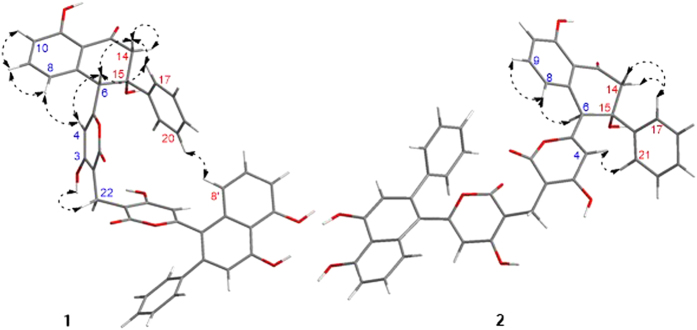
Key NOE correlations for 1 and 2.

**Figure 6:**
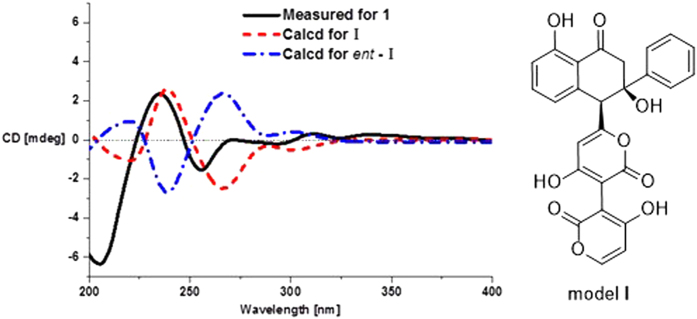
CD and ECD curves of 1, I and ent-I.

Wailupemycin H (**1**) was obtained as yellow, amorphous powder. Its molecular formula was determined as C_43_H_30_O_11_ by the HRESIMS peak at *m/z* 723.1847 [M + H]^+^. Analysis of the ^1^H and ^13^C NMR data (Table 1) indicated that **1** contained α-pyrone and monosubstituted benzene residues similar to those of wailupemycins^9^,^10^,^14^,^15^,^16^. Careful comparison of the 1D (Table 1) and 2D NMR (Fig. 4) data with those of reported wailupemycins suggested the presence of two polyketide subunits A and B (Fig. 1), that is wailupemycins D (**3**)^9^ and G (**5**)^10^ moieties, respectively. However, the two sp^2^-methine signals (*δ*_H/C_ 5.17/89.2, 5.28/89.3) attributed to α-pyrone moieties of wailupemycins D (**3**) and G (**5**) were both disappeared (Table S1). Instead, two relatively downfield sp^2^-quaternary carbon signals (*δ*_C_ 100.2, 100.5) were present (Table 1, Figures S1−S3). In addition, a methylene signals at *δ*_H/C_3.27/17.9 were observed in the NMR spectral of **1**. These data suggested that subunits A and B in **1** were linked together by a methylene group. This deduction was confirmed by the key HMBC correlations of H-22 (*δ*_H_ 3.27) to C-1 (*δ*_C_ 164.9), C-2 (*δ*_C_ 100.2), C-1′ (*δ*_C_ 164.7), and C-2′ (*δ*_C_ 100.5) (Fig. 4). Thus, the constitution of wailupemycin H (**1**) was identified. The relative configuration of **1** was established by NOESY experiment (Fig. 5). Key NOE correlations from both H-14a (*δ*_H_ 3.63) and H-17 (*δ*_H_ 7.48) to H-6 (*δ*_H_ 4.71) and from H-17 (*δ*_H_ 7.48) to H-14a (*δ*_H_ 3.63) indicated the *anti*-orientation of the pyrone and phenyl moieties, suggesting the same relative configuration as **3**^9^. The absolute configuration of **1** (6*S*, 15*R*) was determined by ECD calculations of the simplified model compounds **I** (6*S*, 15*R*) and *ent*-**I** (6*R*, 15*S*) using TD-DFT method at the B3LYP/6-31G(d) level^8^. The results showed that the measured CD curve of **1** is matched well with the calculated ECD for **I** and opposite to that of *ent*-**I** (Fig. 6).

**Figure 7:**
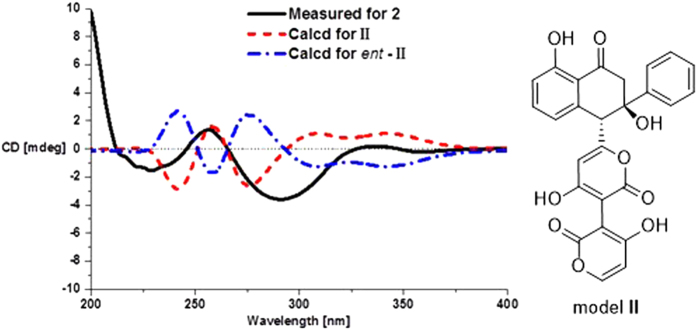
CD and ECD curves of 2, II and *ent*-II.

The molecular formula of wailupemycin I (**2**) was also C_43_H_30_O_11_ from the HRESIMS peak at *m/z* 723.1847 [M + H]^+^, indicating an isomer of **1**. The similar UV, IR and 1D NMR spectra (Table 1) and 2D NMR pattern (Fig. 4) to those of **1** suggested that **2** was a stereoisomer of **1**. The obvious chemical shifts of positions 4–8, 14, 15, and 22 between **1** and **2** further indicated the stereoisomer at C-6 and C-15. The NOESY spectrum (Fig. 5) of **2** showed cross-peaks between H-6 (*δ*_H_ 4.28) and HO-15 (*δ*_H_ 6.07), and between H-4 (*δ*_H_ 5.67) and H-21 (*δ*_H_ 7.39), indicating the same *cis*-orientation of the pyrone and phenyl moieties as wailupemycin E (**4**)^10^. However, no NOESY correlation between H-6 (*δ*_H_ 4.28) and H-14a (*δ*_H_ 2.92) indicated a 6-epimer. This deduction was confirmed by ECD calculations of the simplified model compounds **II** (6*R*, 15*R*) and *ent*-**II** (6*S*, 15*S*) using TD-DFT method at the B3LYP/6-31G(d) level^8^. The consistency of CD curve of **2** to the calculated ECD for **II** and the opposition to ECD of *ent*-**II** (Fig. 7) indicated (6*R*, 15*R*)-configuration of **2.**

**Figure 8:**
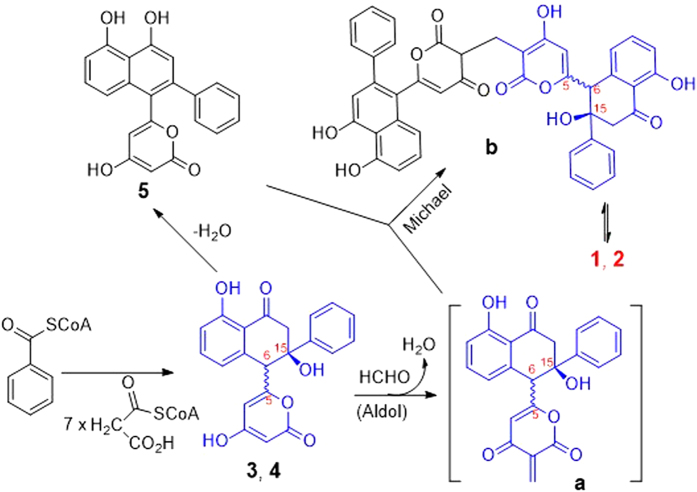
Plausible biosynthetic pathway of 1 and 2.

Compounds **1** and **2** were postulated to be biosynthesized from compounds **3**, **4** and **5** whose biosynthetic pathway had been elucidated to be from benzoyl-CoA and malonyl-CoA^9^,^10^. The aldol condensation took place between **3** or its enolate anion and formaldehyde to produce the key conjugated enone intermediate (**a**). The intermediate **a** further reacted with **5** or its enolate anion *via* a Michael addition to yield the keto-tautomer (**b**) of **1** that formed the more favorable enol-tautomer **1**. By the same procedure, the bio-reactions between compounds **4** and **5** produced compound **2** (Fig. 8).

To elucidate the postulation and to further identify the structures of **1** and **2**, a chemical transformation was performed using compounds **3**, **4** and **5** as the materials. When reacted with **5** and HCHO in EtOH, compounds **3** and **4** formed **1** and **2** that were identified by ESIMS and co-HPLC experiments, respectively (Figure S31).

**Figure 9:**
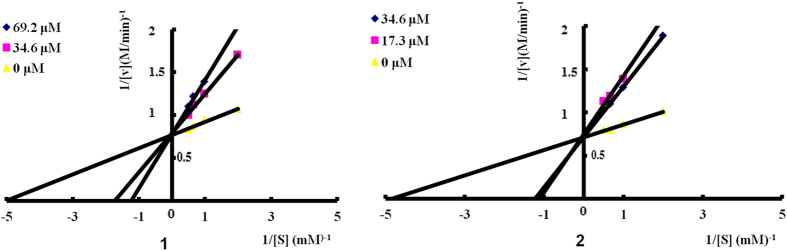
Lineweaver–Burk plot of α-glucosidase inhibition of compounds 1 and 2 (α-glucosidase was treated with pNPG at various concentrations (0.5–2.0 mM) in the absence or presence of 1 and 2 at two different concentrations (34.6 and 69.2 *μ*M for 1 and 17.3 and 34.6 *μ*M for 2). The kinetics assay has been performed after incubating the mixture at 37 °C for 15 min).

Compounds **1**–**5** were assayed for their α-glucosidase inhibitory effects using *p*-nitrophenyl-α-D-glucopyranoside (pNPG) as a substrate^17^,^18^,^19^, and cytotoxicity on murine small intestinal IEC-6 cell line by MTT method^20^ using the acarbose as the positive control. Compounds **1**–**5** exhibited stronger inhibitions of α-glucosidase and lower cytotoxicity than acarbose with the IC_50_/CC_50_ values of 19.7/279.8, 8.3/1317.2, 988.7/2750.0, 392.5/2975.3, and 239.3/2953.8, respectively (IC_50_ for acarbose, 1115.2 *μ*M). The selectivity indexes (SI) of compounds **1**–**5** were 14.2, 158.5, 2.8, 7.6, and 12.3, respectively. The results indicated that the aromatization of cyclohexanone moiety and the 6,15-*cis*-orientation of the pyrone and phenyl moieties tend to increase the α-glucosidase inhibitory activity and decrease the cytotoxicity of these compounds. In addition, a study of enzyme kinetics indicated that **1** and **2** were competitive α-glucosidase inhibitors with the *K*_i_ of 16.8 and 6.0 *μ*M, respectively (Fig. 9).

## Discussion

The aromatic dimers having a methylene linkage are rare in nature, especially originated from microorganism. Most of them were found in plant kingdom, such as italipyrone and homoarenol from *Helichrysum stoechas*^21^, two phloroglucinol derivatives from *H*. *stoechas* var. *olonnense*^22^, helipyrone and norhelipyrone from *H*. *arenarium*^23^, kunzeagin A (a dimeric flavonol glycoside) from *Kunzea ambigua*^24^, gerberinol from *Diospyros kaki* var. *sylvestris*^25^, and four acylphloroglucinol derivatives from *Hypericum andinum*^26^. As far as we know, very few of these natural dimers was identified in microbial kingdom, that is phaeochromycins F from *Streptomyces* sp.^27^, xyloketal F from *Xylaria* sp.^28^, and squarrosidine from *Pholiota squarrosa*^29^. This may be because there was less evidence on the biosynthesis of formaldehyde in microorganisms^30^. The isolation of wailupemycins H and I further reinforced the formaldehyde biosynthetic system could be occurred in microorganisms. And the good α-glucosidase inhibitory activity could also provide the alternative bioactivity screening for this kind of natural dimmers.

## Methods

### General experimental procedures

Optical rotations were measured with a JASCO P-1020 digital polarimeter. UV data were recorded with a Beckman DU 640 spectrophotometer, and CD data were collected using a JASCO J-815 spectropolarimeter. IR spectra were taken on a Nicolet NEXUS 470 spectrophotometer as KBr disks. ^1^H NMR, ^13^C NMR, DEPT, HMQC, HMBC, COSY, and NOESY spectra were recorded using Bruker Avance 500 spectrometer using TMS as an internal standard, and chemical shifts were recorded as *δ* values. Chemical shift values were referenced to residual solvent signals for DMSO (*δ*_H_/*δ*_C_, 2.50/ 39.5). HRESIMS data were recorded using a Q-TOF ULTIMA GLOBAL GAA076 LC mass spectrometer. HPLC and semi-preparative HPLC were performed using a Cholester column (COSMOSIL-pack, 4.6 × 250 mm, 5 *μ*m, 1 mL/min) and an ODS column [YMC-pack ODS-A, 10 × 250 mm, 5 μm, 4 mL/min], respectively. TLC and column chromatography (CC) were performed on plates precoated with silica gel GF254 (10−40 *μ*m) and over silica gel (200−300 mesh, Qingdao Marine Chemical Factory) and Sephadex LH-20 (Amersham Biosciences). Vacuum-liquid chromatography (VLC) used silica gel H (Qingdao Marine Chemical Factory).

### Actinobacterial material

The actinobacterial strain *Streptomyces* sp. OUCMDZ-3434 was isolated from *E*. *prolifera* collected from the Zhanqiao Beach (E 120°18′ 56.982″, N 36°03′ 42.659″, pH 6.0 in sea water), Qingdao, China in July 2012. The *E*. *prolifera* (1 g) were clipped and ground suspending in sterile distilled water. And then serially diluted to 1 mg/mL, 100 *μ*L of which was deposited on a Gause’s synthetic agar plate containing chloramphenicol (100 *μ*g/mL) as a bacterial inhibitor and incubated at 28 °C for 8 days. A single colony was transferred onto another Gause’s synthetic agar plate and was identified according to its morphological characteristics and 16S rRNA gene sequences (GenBank access No. KJ818249). A reference culture is maintained in our lab. at −80 °C. Working stocks were prepared on Gause’s synthetic agar slants and stored at 4 °C.

### Fermentation and extraction

Spores were directly inoculated into 500 mL Erlenmeyer flasks containing 150 mL fermentation media consisted of glucose 20 g, beef extract 3 g, yeast extract 10 g, soluble starch 10 g, peptone 10 g, K_2_HPO_4_ 0.5 g, MgSO_4_ 0.5 g, CaCO_3_ 2 g, and 1 L of old sea water, pH nature). The flasks were incubated on a rotatory shaker at 180 rpm and 28 °C for 8 days. 45 L of whole broth was extracted with equal volumes of EtOAc for three times. The EtOAc extract was concentrated under reduced pressure to give a dark brown gum (28.0 g).

### Purification

The EtOAc extract (28 g) was subjected to a silica gel VLC column, eluting with a stepwise gradient of petroleum ether–CH_2_Cl_2_ (1:1 and 0:1) and then with MeOH–CH_2_Cl_2_ (100:1, 75:1, 50:1, 25:1, 10:1, 1:1) to give eight fractions (Fr1–Fr8). Fr2–Fr5 (4.68 g) were combined and separated on a HP20SS column, eluting with a stepwise gradient of MeOH–H_2_O (0%–100%, v/v) to give six fractions (H1–H8). Fraction H4 (403.5 mg) was subjected to a Sephadex LH-20 column eluting with MeOH to give four fractions (H4-1 to H4-4). Fraction H4-3 (38.1 mg) was further purified by HPLC on ODS (45% MeOH−H_2_O, v/v) to yield **4** (5.6 mg, t_R_13.9 min). Fraction H5 (121.3 mg) was subjected to a Sephadex LH-20 column eluting with MeOH to give three fractions (H5-1 to H5-3). Fraction H5-2 (31.2 mg) was further purified by HPLC on ODS (55% MeOH−H_2_O, v/v) to yield **3** (14.7 mg, t_R_ 10.5 min). Fraction H6 (243.7 mg) was also subjected to a Sephadex LH-20 column eluting with MeOH to give four fractions (H6-1 to H6-4). Fraction H6-2 (73.6 mg) was further purified by HPLC on ODS (70% MeOH−H_2_O, v/v) to yield **5** (12.2 mg, t_R_ 6.5 min). Fraction H6-3 (47.7 mg) was subjected to another Sephadex LH-20 column eluting with MeOH and further purified by HPLC on ODS (80% MeOH−H_2_O, v/v) to yield **1** (4 mg, t_R_ 6.5 min) and **2** (2.9 mg, t_R_ 5.6 min).

#### Wailupemycin H (1)

yellow, amorphous powder; [α]^25^_D_ +18.1 (*c* 0.1, MeOH); UV (MeOH) *λ*_max_ (logε) 208 (4.49), 260 (4.18), 334 (3.82) nm; CD (*c* 0.11, MeOH) *λ*_max_ (Δε) 206 (−1.28), 235 (+0.48), 256 (−0.31), 311 (+0.06) nm; IR (KBr) *ν*_max_ 2922, 1704, 1677, 1571, 1198, 699 cm^−1^; ^1^H and ^13^C NMR data, see Table 1; HRESIMS *m/z* 723.1847 [M + H]^+^ (calcd for C_43_H_31_O_11_, 723.1861).

#### Wailupemycin I (2)

yellow, amorphous powder; [α]^25^_D_ −39.6 (*c* 0.1, MeOH); UV (MeOH) *λ*_max_ (logε) 208 (4.49), 260 (4.18), 334 (3.82) nm; CD (*c* 0.11, MeOH) *λ*_max_ (Δε) 229 (−0.31), 257 (+0.28), 291 (−0.73), 337 (+0.04) nm; IR(KBr) *ν*_max_ 2922, 1704, 1677, 1571, 1198, 699 cm^−1^; ^1^H and ^13^C NMR data, see Table 1; HRESIMS *m/z* 723.1847 [M + H]^+^ (calcd for C_43_H_31_O_11_, 723.1861).

#### Wailupemycin D (3)

yellow, amorphous powder; [α]^25^_D_ +16.4 (*c* 0.1, MeOH); UV (MeOH) *λ*_max_ (logε) 216 (3.53), 256 (3.12), 333 (1.57) nm; CD (*c* 0.73, MeOH) *λ*_max_ (Δε) 212 (+0.1), 224 (+0.97), 263 (−0.2), 289 (+0.57) nm; ^1^H and ^13^C NMR data, see Table S1. ESI-MS *m/z* 365.2 [M + H]^+^.

#### Wailupemycin E (4)

yellow, amorphous powder; [α]^25^_D_ −28.6 (*c* 0.1, MeOH); UV (MeOH) *λ*_max_ (logε) 215 (3.51), 256 (3.23), 332 (1.48) nm; CD (*c* 0.73, MeOH) *λ*_max_ (Δε) 231 (−0.73), 258 (+0.5), 285 (−1.31), 329 (+0.11)nm; ^1^H and ^13^C NMR data, see Table S1. ESI-MS *m/z* 365.2 [M + H]^+^.

### Horeau’s experiment

To a solution of **3** (5 mg, 13.7 *μ*mol) in dry pyridine(1.5 mL), racemic 2-phenylbutyryl chloride (15 *μ*L, 90.7 *μ*mol) was added. The reaction mixture was stirred at r.t. for 36 h. Water (3.0 mL) was then added and the mixture was allowed to stand for 30 min. The solution was extracted with EtOAc (3 × 10 mL) after adjusting the pH value to 9 by dropwise addition of NaOH (0.1 *M*). The aqueous layer was acidified to pH 3 using HCl (1 *M*) and then extracted with CH_2_Cl_2_ (3 × 10 mL). Evaporation of the CH_2_Cl_2_ solution gave the unreacted 2-PHA (5.3 mg) with [α]^25^_D_ +5.8 (*c* 0.35, MeOH), indicating the *S*- enantiomeric excess.

### Chemical transformations

To a solution of **3** (1.1 mg, 2.89 *μ*mol) and **5** (1 mg, 2.89 *μ*mol) in EtOH (1 mL) was added 1.85% formaldehyde solution (4 *μ*L, 2.89 *μ*mol) that was prepared by dilution of 37% HCHO aqueous solution with EtOH. The reaction mixture was heated at 80 °C for 3 h. HPLC analysis revealed three products one of which was identified as **1** by ESIMS peak at *m/z* 723.3 [M + H]^+^ and co-HPLC with the natural-**1** (t_*R*_ 5.21 min, 80% MeCN/H_2_O, cholester packed column) (Figure S31). By the same procedure, compound **2** was formed from the reaction of **4** (1.1 mg, 2.89 *μ*mol) with **5** (1 mg, 2.89 *μ*mol) and 1.85% formaldehyde (4 *μ*L, 2.89 *μ*mol), and was identified by co-HPLC with the natural-**2** (t_*R*_ 5.05 min, 80% MeCN/H_2_O, cholester packed column) along with the ESIMS peak at *m/z* 723.2 [M + H]^+^ (Figure S31).

### α-Glucosidase inhibitory effect assay

The inhibitory effects were assayed as described preciously^17^. The sample was dissolved in sodium phosphate buffer (PBS, pH 6.8) at three concentrations. A volume of 10 *μ*L of the sample solution, 20 *μ*L of PBS and 20 *μ*L of 2.0 mm *p*-nitrophenyl-α-D-glucopyranoside (pNPG) solution (in phosphate buffer) were mixed in a 96-well microplate and incubated at 37 °C for 5 min. A volume of 10 *μ*L of α-glucosidase diluted to 0.2 U/mL by 0.01 M PBS was then added to each well. After incubating at 37 °C for 15 min, the absorbance at 405 nm was recorded by a Spectra max 190 micro plate reader (Molecular Devices Inc.). The blank was prepared by adding phosphate buffer instead of the α-glucosidase and the acarbose was used as a positive control. Blank readings (no enzyme) were subtracted from each well and results were compared to the control. The inhibition (%) was calculated as [1 − (OD_drug_/OD_blank_)] ×100%. The IC_50_ value was calculated as the compound concentration that is required for 50% inhibition and the IC_50_ value of the acarbose was 1.12 mM.

### Kinetics of α-glucosidase inhibitors

According to the paper^18^,^19^, the mode of inhibition of compounds **1** and **2** against α-glucosidase activity was measured with increasing concentrations of pNPG (0, 0.5, 1, 1.5 and 2.0 mM) as a substrate in the absence and presence of **1** and **2** at 34.6 and 69.2 *μ*M, and 17.3 and 34.6 *μ*M, respectively. Optimal amounts of compounds **1** and **2** used were determined based on the enzyme inhibitory activity assay. Mode of inhibition of **1** and **2** were determined by Lineweaver-Burk plot analysis of the data that were calculated by Michaelis-Menten kinetics.

### Cytotoxic assay

The cytotoxicity on murine small intestinal IEC-6 cells were assayed by MTT (3-[4,5-dimethyl thiazol-2-yl]-2,5-diphenyl tetrazolium bromide) method^20^. The IEC-6 cells were cultured in Dulbecco’s modified Eagle’s medium (DMEM) supplemented with 5% fetal bovine serum (FBS) under a humidified atmosphere of 5% CO_2_ and 95% air at 37 °C. Cell suspension, 100 *μ*L, at a density of 2 × 10^4^ cell mL^−1^ was plated in 96-well microtiter plates and and exposed to different concentrations of compounds in triplicate for 72 h. The initial concentrations of compounds were 2 mg/mL in DMSO, and then were diluted into 625, 125, 25, 5, and 1 *μ*g/mL with RPMI-1640 medium, respectively. The experiments were divided into blank control, testing compounds and the positive control groups, and each group was set up three parallels. Then, 10 *μ*L of PBS containing MTT (final concentration: 0.5 mg/mL) was added to each well. After 4 h incubation at 37 °C, the supernatant was removed and 200 *μ*L of DMSO was added to each well to solubilize the formazan crystals. After vigorous shaking, absorbance values were measured in a microplate reader (Bio-Rad, USA) at 570 nm. The cytotoxicity was represented at these concentrations as [(OD_blank_ − OD_drug_)/OD_blank_ × 100%]. The CC_50_ value, defined as the compound concentration necessary to induce cell cytotoxicity by 50%, was calculated by SPSS (Statistical Product and Service Solutions) v19.0 software.

### ECD calculations

The calculations were performed by using the density functional theory (DFT) as carried out in the Gaussian 03^30^. The preliminary conformational distributions search was performed by HyperChem 7.5 software. All ground-state geometries were optimized at the B3LYP/6-31G(d) level. Solvent effects of methanol solution were evaluated at the same DFT level by using the SCRF/PCM method^31^,^32^,^33^. TDDFT^34^,^35^,^36^,^37^ at B3LYP/6-31G(d) was employed to calculate the electronic excitation energies and rotational strengths in methanol. The stable conformations obtained at the B3LYP/6-31G(d) level were further used in magnetic shielding constants at the B3LYP/6-311++G(2d,p) level.

## Additional Information

**How to cite this article**: Chen, Z. *et al.* New α-glucosidase inhibitors from marine algae-derived *streptomyces* sp. OUCMDZ-3434. *Sci. Rep.* **6**, 20004; doi: 10.1038/srep20004 (2016).

## Supplementary Material

Supplementary Information

## Figures and Tables

**Table 1:** ^1^H (500 MHz) and ^13^C (125 MHz) NMR Data for **1** and **2** in DMSO-*d*_*6*_ (TMS, *δ* ppm)^a^.

Position	1	*δ*_H_,(*J* in Hz)	2	*δ*_H_ (*J* in Hz)
	*δ*_C_		*δ*_C_	
1 (C)	164.9		164.8	
2 (C)	100.2		99.9	
3 (C)	173.3		173.5	
4 (CH)	104.4	6.18, s	103.3	5.67, s
5 (C)	160.5		159.6	
6 (CH)	54.1	4.71, s	55.3	4.28, s
7 (C)	142.5		141.9	
8 (CH)	119.4	6.58, d (8.3)	120.7	6.88, d (8.3)
9 (CH)	137.1	7.50, t (7.6)	137.0	7.53, t (7.6)
10 (CH)	116.2	6.89, d (8.3)	116.3	6.90, d (8.3)
11 (C)	161.5		161.4	
12 (C)	116.1		116.0	
13 (C)	203.7		204.4	
14 (CH_2_)	51.2	3.03, d (17.0) 3.63, d (17.0)	45.9	2.92, d (17.2) 3.94, d (17.2)
15 (C)	75.4		74.8	
16 (C)	144.9		144.4	
17/21 (CH)	125.4	7.48, m	125.2	7.39, m
18/20 (CH)	127.2	7.34, m	127.5	7.31, m
19 (CH)	127.6	7.23, m	127.6	7.25, m
1′ (C)	164.7		163.9	
2′ (C)	100.5		100.3	
3′ (C)	173.0		173.2	
4′ (CH)	106.2	5.80, s	106.7	5.75, s
5′ (C)	157.5		157.5	
6′ (C)	119.5		119.5	
7′ (C)	135.2		135.2	
8′ (CH)	116.3	7.04, d (8.3)	116.3	6.99, d (8.3)
9′ (CH)	128.7	7.37, m	128.7	7.37, m
10′ (CH)	109.3	6.84, d (8.3)	109.3	6.83, d (8.3)
11′ (C)	154.5		154.5	
12′ (C)	113.4		113.4	
13′ (C)	155.8		155.8	
14′ (CH)	109.8	6.78, s	109.8	6.77, s
15′ (C)	140.2		140.2	
16′ (C)	141.0		141.0	
17′/21′ (CH)	128.5	7.25, m	128.5	7.26, m
18′/20′ (CH)	128.0	7.33, m	128.1	7.32, m
19′ (CH)	128.2	7.24, m	128.3	7.25, m
22 (CH_2_)	17.9	3.27, s	17.8	3.15, s

^a^The proton signals of HO-3, HO-11, HO-15, HO-3′ for **1** were 11.43 (br.s), 12.39 (br.s), 6.05 (br.s), and 11.30 (br.s), respectively. And the proton signals of HO-3, HO-11, and HO-15 for **2** were 11.33 (br.s), 12.36 (br.s), and 6.07 (br.s), respectively. However, the proton signals for HO-11′ and HO-13′ of **1** and HO-3′, HO-11′ and HO-13′ of **2** were not observed.
