# Supplementary material for: New α-glucosidase inhibitors from marine algae-derived Streptomyces sp. OUCMDZ-3434
Source: Sci Rep. 2016 Jan 29;6:20004. doi: 10.1038/srep20004 (PMC4731795; doi:10.1038/srep20004)

Supplementary Information

New *α*-glucosidase inhibitors from marine algae-derived *Streptomyces* sp. OUCMDZ-3434

Zhengbo Chen[1], Jiejie Hao[1], Liping Wang[2], Yi Wang[1,*], Fandong Kong[1] and Weiming Zhu[1,*]

1. Key Laboratory of Marine Drugs, Ministry of Education of China, School of Medicine and Pharmacy, Ocean University of China, Qingdao 266003, China

2. Key Laboratory of Chemistry for Natural Products of Guizhou Province and Chinese Academy of Sciences, Guiyang 550002, China

* Correspondence. weimingzhu@ouc.edu.cn, wangyi0213@ouc.edu.cn.

List of Supplementary Information

16S rRNA gene sequences and the phylogenetic tree of *Streptomyces* sp. OUCMDZ-3434 2

Table S1. ^{1}H and ^{13}C NMR Data for **3**–**5** (500, 125 MHz, DMSO-d_6, TMS, δ ppm).. 4

Figure S1. The ^{1}H-NMR spectrum of wailupemycin H (**1**) in DMSO-d_6...5

Figure S2. The ^{13}C-NMR spectrum of wailupemycin H (**1**) in DMSO-d_6 6

Figure S3. The DEPT spectrum of wailupemycin H (**1**) in DMSO-d_6 7

Figure S4. The HMQC spectrum of wailupemycin H (**1**) in DMSO-d_6..8

Figure S5. The enlarged HMQC spectrum of wailupemycin H (**1**) in DMSO-d_6 9

Figure S6. The ^{1}H-^{1}H COSY spectrum of wailupemycin H (**1**) in DMSO-d_6...10

Figure S7. The enlarged ^{1}H-^{1}H COSY spectrum of wailupemycin H (**1**) in DMSO-d_6 (δ 6.40-7.60) 11

Figure S8. The HMBC spectrum of wailupemycin H (**1**) in DMSO-d_6 12

Figure S9. The enlarged HMBC spectrum of wailupemycin H (**1**) in DMSO-d_6 (δ 5.70-7.60)........................13

Figure S10. The enlarged HMBC spectrum of wailupemycin H (**1**) in DMSO-d_6 (δ 3.10-4.80) 14

Figure S11. The NOSEY spectrum of wailupemycin H (**1**) in DMSO-d_6..15

Figure S12. The ^{1}H-NMR spectrum of wailupemycin I (**2**) in DMSO-d_6 16

Figure S13. The ^{13}C-NMR spectrum of wailupemycin I (**2**) in DMSO-d_6..17

Figure S14. The DEPT spectrum of wailupemycin I (**2**) in DMSO-d_6...18

Figure S15. The HMQC spectrum of wailupemycin I (**2**) in DMSO-d_6..19

Figure S16. The enlarged HMQC spectrum of wailupemycin I (**2**) in DMSO-d_6 (δ 5.40-7.90) 20

Figure S17. The ^{1}H-^{1}H COSY spectrum of wailupemycin I (**2**) in DMSO-d_6 21

Figure S18. The enlarged ^{1}H-^{1}H COSY spectrum of wailupemycin I (**2**) in DMSO-d_6 (δ 6.65-7.65) 22

Figure S19. The HMBC spectrum of wailupemycin I (**2**) in DMSO-d_6..23

Figure S20. The enlarged HMBC spectrum of wailupemycin I (**2**) in DMSO-d_6 (δ 5.40-7.70)........................24

Figure S21. The enlarged HMBC spectrum of wailupemycin I(**2**) in DMSO-d_6 (δ 3.10-4.60)…………………….....25
Figure S22. The NOSEY spectrum of wailupemycin I (**2**) in DMSO-d_6……………………………………………...26
Figure S23. The ^{1}H-NMR spectrum of wailupemycin D (**3**) in DMSO-d_6……………………………………………27
Figure S24. The ^{13}C-NMR spectrum of wailupemycin D (**3**) in DMSO-d_6 ..…………………………………………28
Figure S25. The DEPT spectrum of wailupemycin D (**3**) in DMSO-d_6 ……………………………………………29
Figure S26. The NOSEY spectrum of wailupemycin D (**3**) in DMSO-d_6……………………………………………30
Figure S27. The ^{1}H-NMR spectrum of wailupemycin E (**4**) in DMSO-d_6……………………………………………31
Figure S28. The ^{13}C-NMR spectrum of wailupemycin E (**4**) in DMSO-d_6..…………………………………………32
Figure S29. The DEPT spectrum of wailupemycin E (**4**) in DMSO-d_6.……………………………………………33
Figure S30. The NOSEY spectrum of wailupemycin E (**4**) in DMSO-d_6 ……………………………………………34
Figure S31. ESI-MS, HPLC and co-HPLC profiles of the synthetic **1** and **2** with the natural ones ……………35
Table S2. Energy calculation for stable conformers of **3a** and **3b** ……………………………………………………36

16S rRNA gene sequences and the phylogenetic tree of *Streptomyces* sp. OUCMDZ-3434 （GenBank No. KJ818249）

GCGTGCTTACACATGCAAGTCGAACGATGAACCGCTTTCGGGCGGGGATTAGTGGCGAACGGGTGAGTAACACGTGGGC
AATCTGCCCTGCACTCTGGGACAAGCCCTGGAAACGGGGTCTAATACCGGATATGACCGTCTGCCGCATGGTGGATGGTG
TAAAGCTCCGGCGGTGCAGGATGAGCCCGCGGCCTATCAGCTTGTTGGTGAGGTAGTGGCTCACCAAGGCGACGACGGG
TAGCCGGCCTGAGAGGGCGACCGGCCACACTGGGACTGAGACACGGCCCAGACTCCTACGGGAGGCAGCAGTGGGGA
ATATTGCACAATGGGCGAAAGCCTGATGCAGCGACGCCGCGTGAGGGATGACGGCCTTCGGGTTGTAAACCTCTTTCAG
CAGGGAAGAAGCGAAAGTGACGGTACCTGCAGAAGAAGCGCCGGCTAACTACGTGCCAGCAGCCGCGGTAATACGTAG
GGCGCAAGCGTTGTCCGGAATTATTGGGCGTAAAGAGCTCGTAGGCGGCTTGTCACGTCGGTTGTGAAAGCCCGGGGCT
TAACCCCGGGTCTGCAGTCGATACGGGCAGGCTAGAGTTCGGTAGGGGAGATCGGAATTCCTGGTGTAGCGGTGAAATG
CGCAGATATCAGGAGGAACACCGGTGGCGAAGGCGGATCTCTGGGCCGATACTGACGCTGAGGAGCGAAAGCGTGGGG
AGCGAACAGGATTAGATACCCTGGTAGTCCACGCCGTAAACGGTGGGCACTAGGTGTGGGCAACATTCCACGTTGTCCG
TGCCGCAGCTAACGCATTAAGTGCCCCGCCTGGGGAGTACGGCCGCAAGGCTAAAACTCAAAGGAATTGACGGGGGCC
CGCACAAGCGGCGGAGCATGTGGCTTAATTCGACGCAACGCGAAGAACCTTACCAAGGCTTGACATACACCGGAAACGT
CTGGAGACAGGCGCCCCCTTGTGGTCGGTGTACAGGTGGTGCATGGCTGTCGTCAGCTCGTGTCGTGAGATGTTGGGTT
AAGTCCCGCAACGAGCGCAACCCTTGTCCCGTGTTGCCAGCAGGCCCTTGTGGTGCTGGGGACTCACGGGAGACCGCC
GGGGTCAACTCGGAGGAAGGTGGGGACGACGTCAAGTCATCATGCCCCTTATGTCTTGGGCTGCACACGTGCTACAATG
GCCGGTACAATGAGCTGCGATACCGTGAGGTGGAGCGAATCTCAAAAAGCCGGTCTCAGTTCGGATTGGGGTCTGCAAC
TCGACCCCATGAAGTCGGAGTCGCTAGTAATCGCAGATCAGCATTGCTGCGGTGAATACGTTCCCGGGCCTTGTACACAC
CGCCCGTCACGTCACGAAAGTCGGTAACACCCGAAGCCGGTGGCCCAACCCCTTGCTGGGAGGGAGCTGTCGAAGGTG
GGACTGGCGATTGGGACGAAGTCGTAACAAGTAGCC

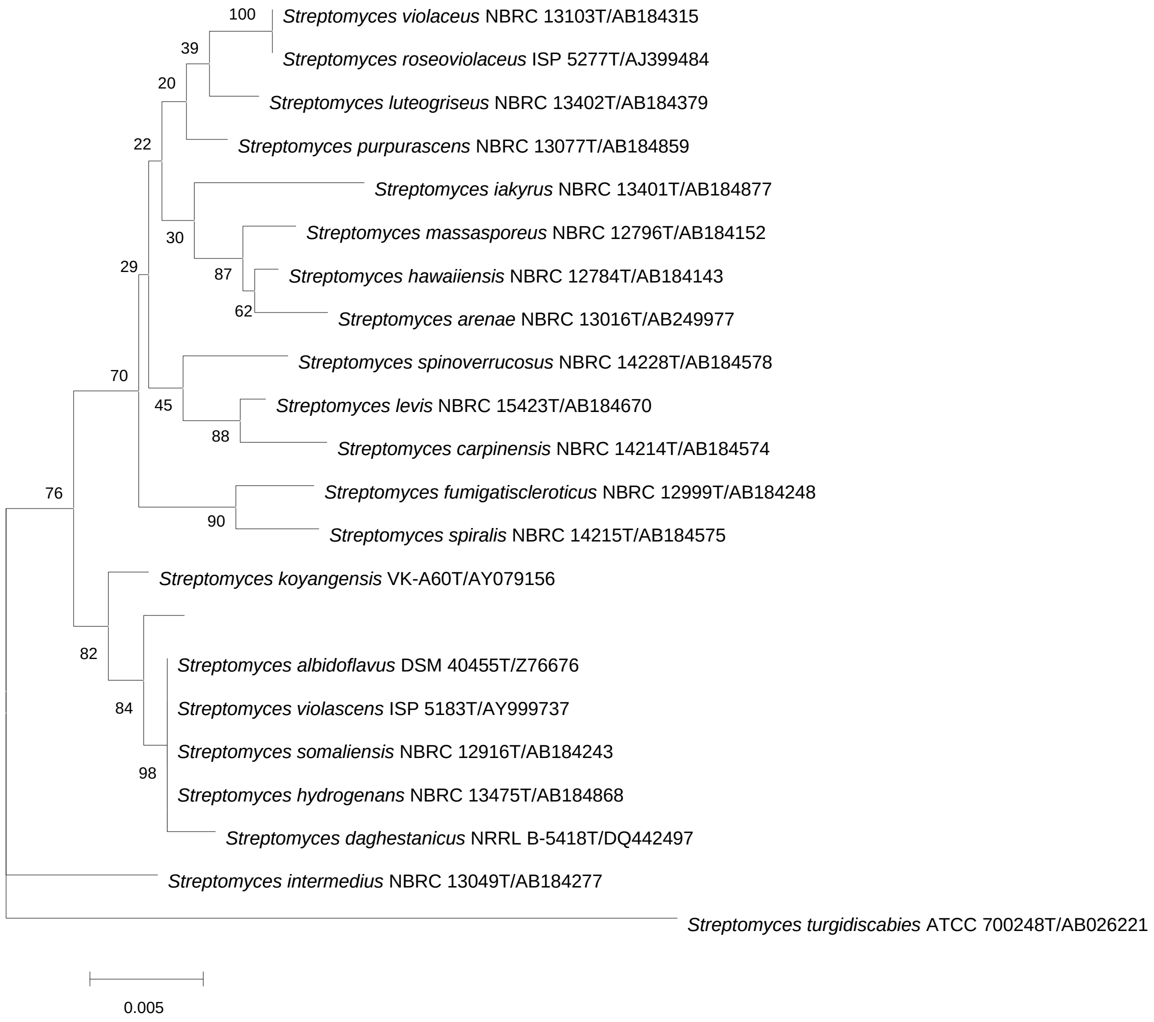

Neighbor-joining phylogenetic tree of strain OUCMDZ-3434 based on 16S rRNA gene sequences (ca. 1453bp). The values at each node represent the bootstrap values from 1000 replicates, and the scale bar represents 0.005 substitutions per nucleotide. Phylogenetic analyses were conducted in MEGA4.

Table S1. ^{1}H and ^{13}C NMR Data for compounds 3-5 (500, 125 MHz, DMSO-d_6, TMS, δ in ppm)

Position	3		4		5	
	δ_C	δ_H (J in Hz)	δ_C	δ_H (J in Hz)	δ_C	δ_H (J in Hz)
1	164.0, C		162.9, C		163.9, C	
2	89.2, CH	5.17, d (2)	89.0, CH	5.01, d (2)	89.3, CH	5.28, d (2)
3	169.9, C		169.9, C		169.9, C	
4	104.0, CH	6.13, s	102.8, CH	5.69, d (2)	105.8, CH	5.83, d (2)
5	163.2, C		163.0, C		161.0, C	
6	54.3, CH	4.74, s	55.3, CH	4.34, s	119.5, C	
7	142.2, C		141.5, C		135.0, C	
8	119.3, CH	6.59, d (7.5)	120.6, CH	6.85, d (7.5)	116.1, CH	7.09, d (7.5)
9	137.1, CH	7.51, t (7.5)	136.7, CH	7.49, t (7.5)	128.8, CH	7.39, m
10	116.3, CH	6.89, d (7.5)	116.3, CH	6.7, d (8)	109.3, CH	6.85, d (8)
11	161.5, C		161.4, C		154.5, C	
12	116.2, C		116.0, C		113.4, C	
13	203.6, C		204.3, C		155.8, C	
14	50.9, CH_2	3.04, d (17), 3.66, d (17)	45.8, CH_2	2.90, d (17), 3.92, t (17)	109.8, CH	6.78, s
15	75.5, CH		74.6, CH		140.2, C	
16	144.9, C		144.4, C		141.1, C	
17/21	125.5, CH	7.49, d (7.5)	125.2, CH	7.38, d (7.5)	128.5, CH	7.36, d (7.40)
18/20	127.3, CH	7.31, t (7.5)	127.7, CH	7.31, t (7.5)	128.3, CH	7.30, m
19	128.0, CH	7.22, t (7.5)	128.1, CH	7.24, t (7.5)	128.4, CH	7.33, m
3-HO		11.65, br.s		11.59, br.s		11.5, br.s
11-HO		12.39, br.s		12.30, br.s		
15-HO		6.02, br.s		6.05, br.s		

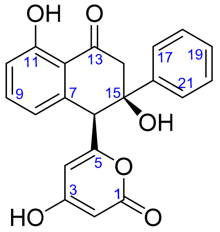

3

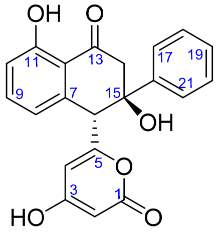

4

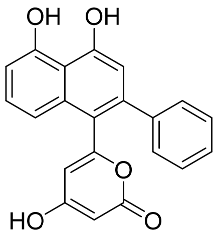

5

Figure S1. The ^{1}H-NMR spectrum of wailupemycin H (1) in DMSO-d_6

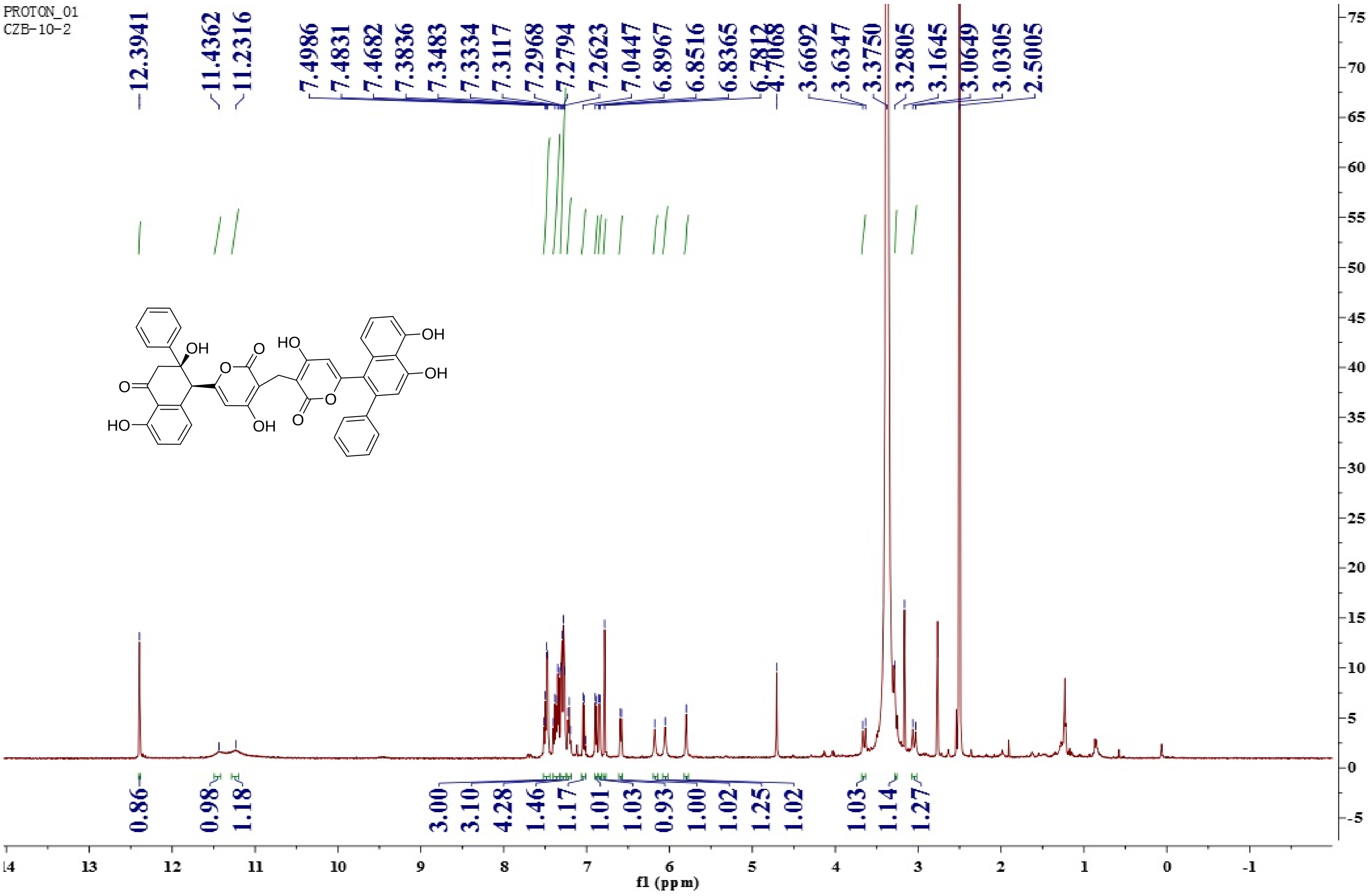

Figure S2. The ^{13}C-NMR spectrum of wailupemycin H (1) in DMSO-d_6

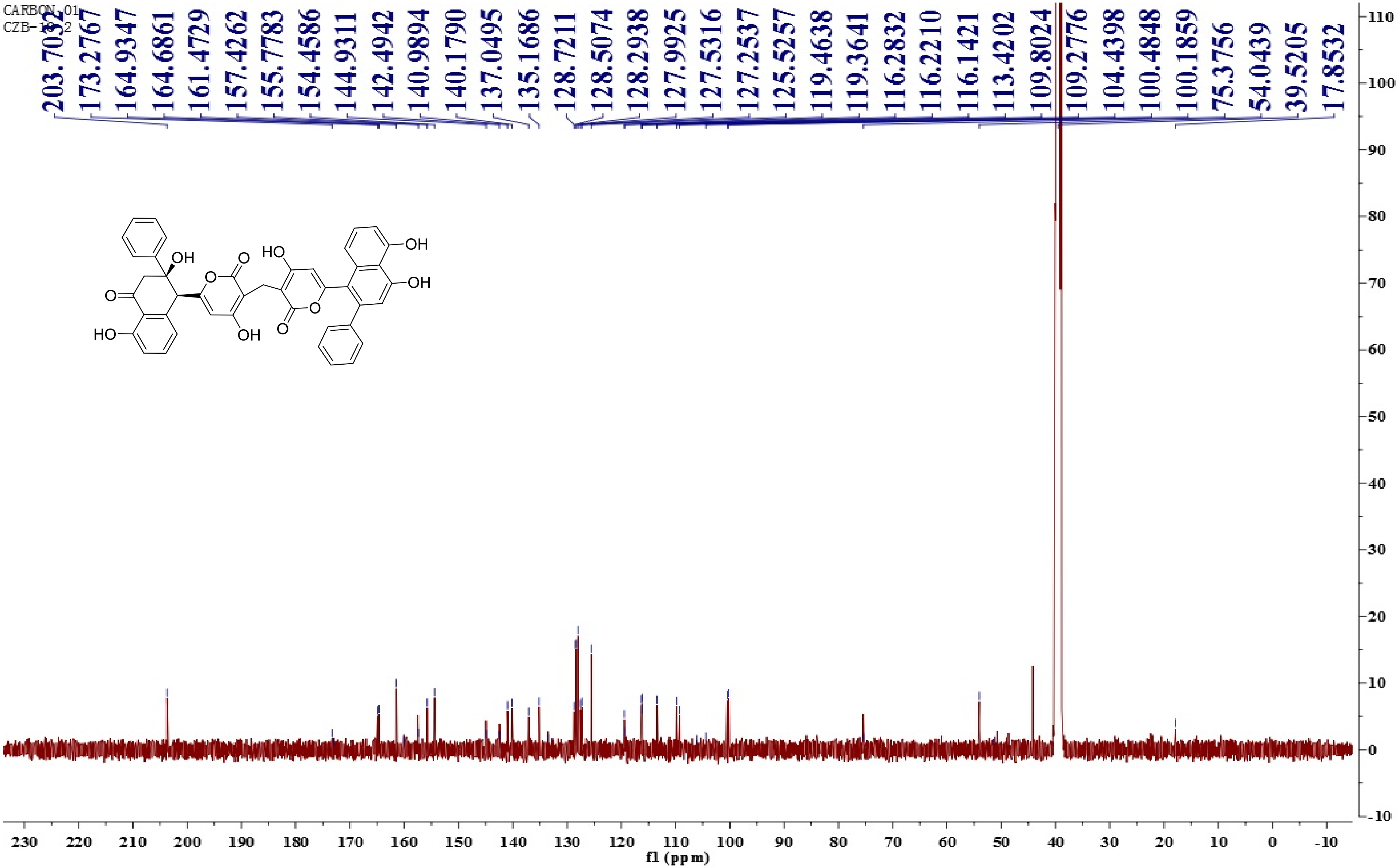

Figure S3. The DEPT spectrum of wailupemycin H (1) in DMSO-d_6

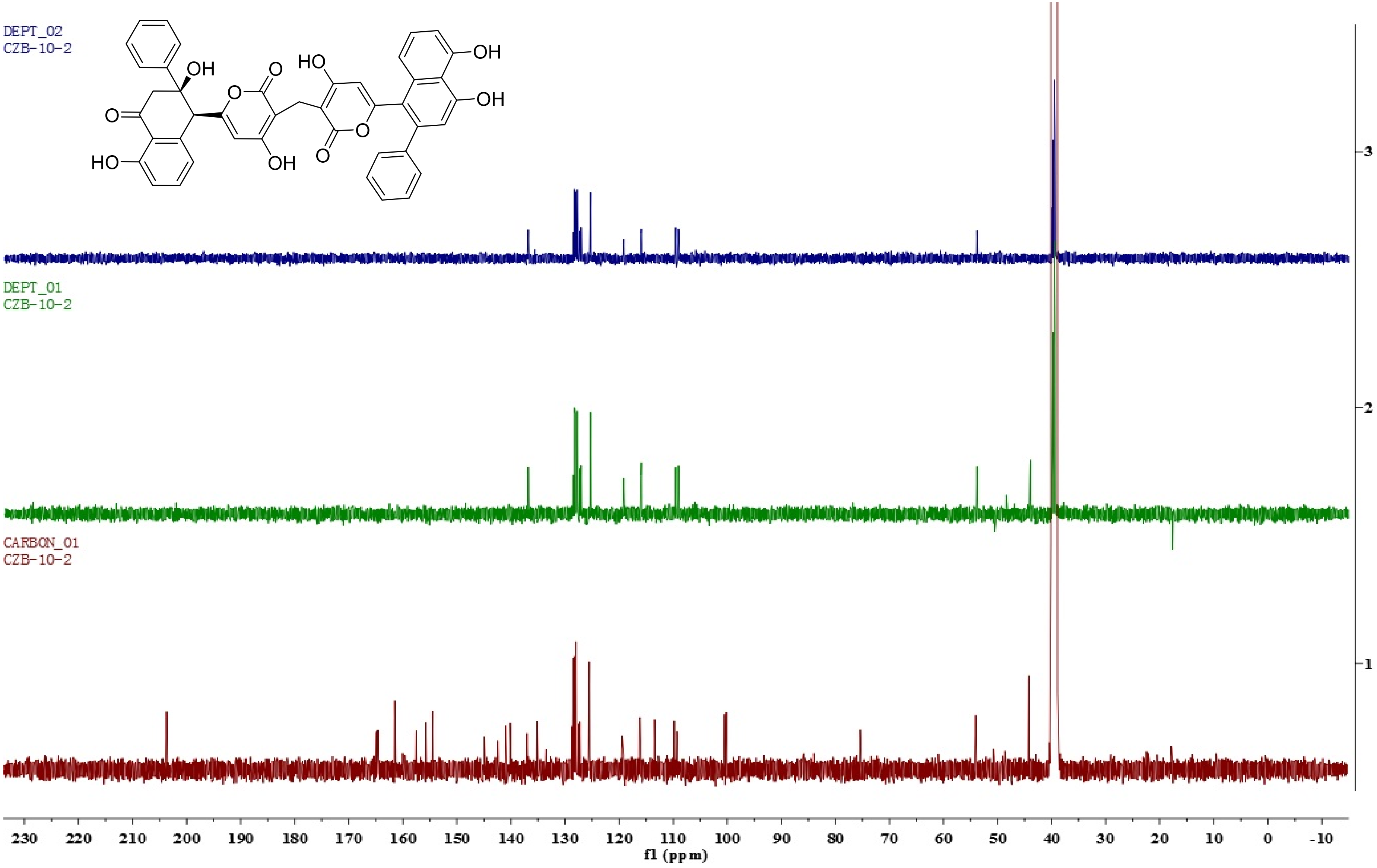

Figure S4. The HMQC spectrum of wailupemycin H(1) in DMSO-d_6

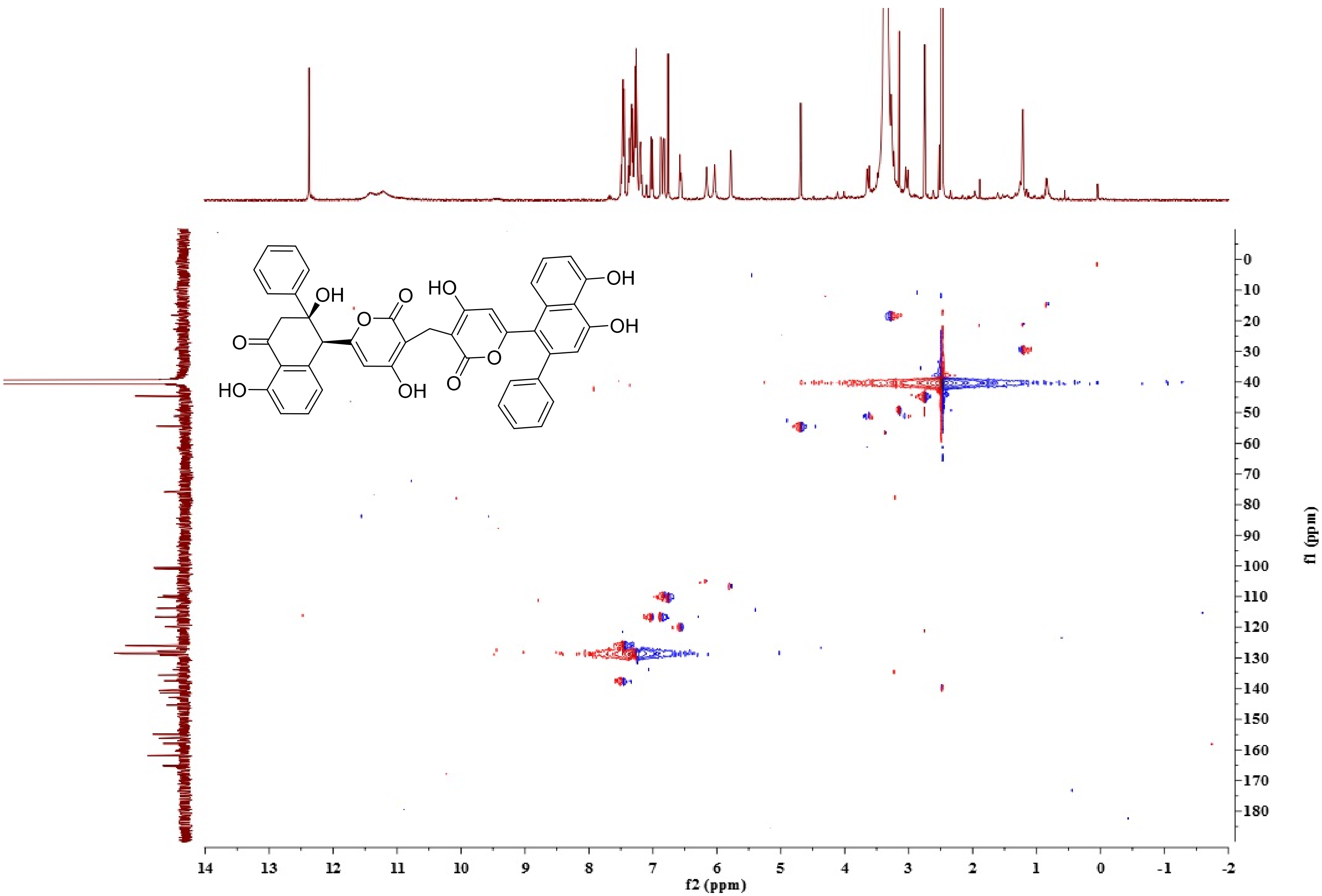

Figure S5. The enlarged HMQC spectrum of wailupemycin H (1) in DMSO-d_6 (δ_H 5.60- δ_H 7.60)

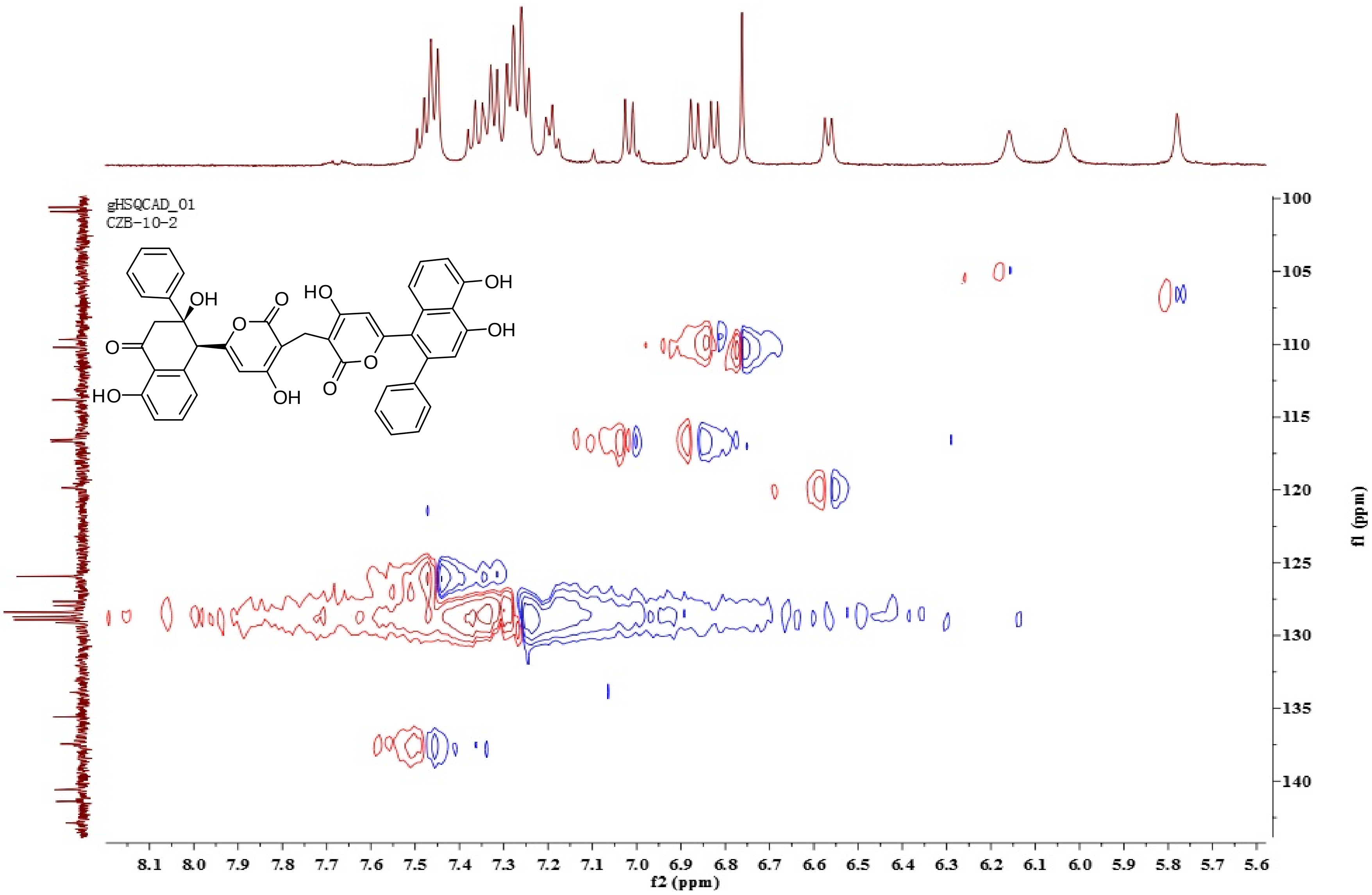

Figure S6. The ^1H-^1H COSY spectrum of wailupemycin H (1) in DMSO-d_6

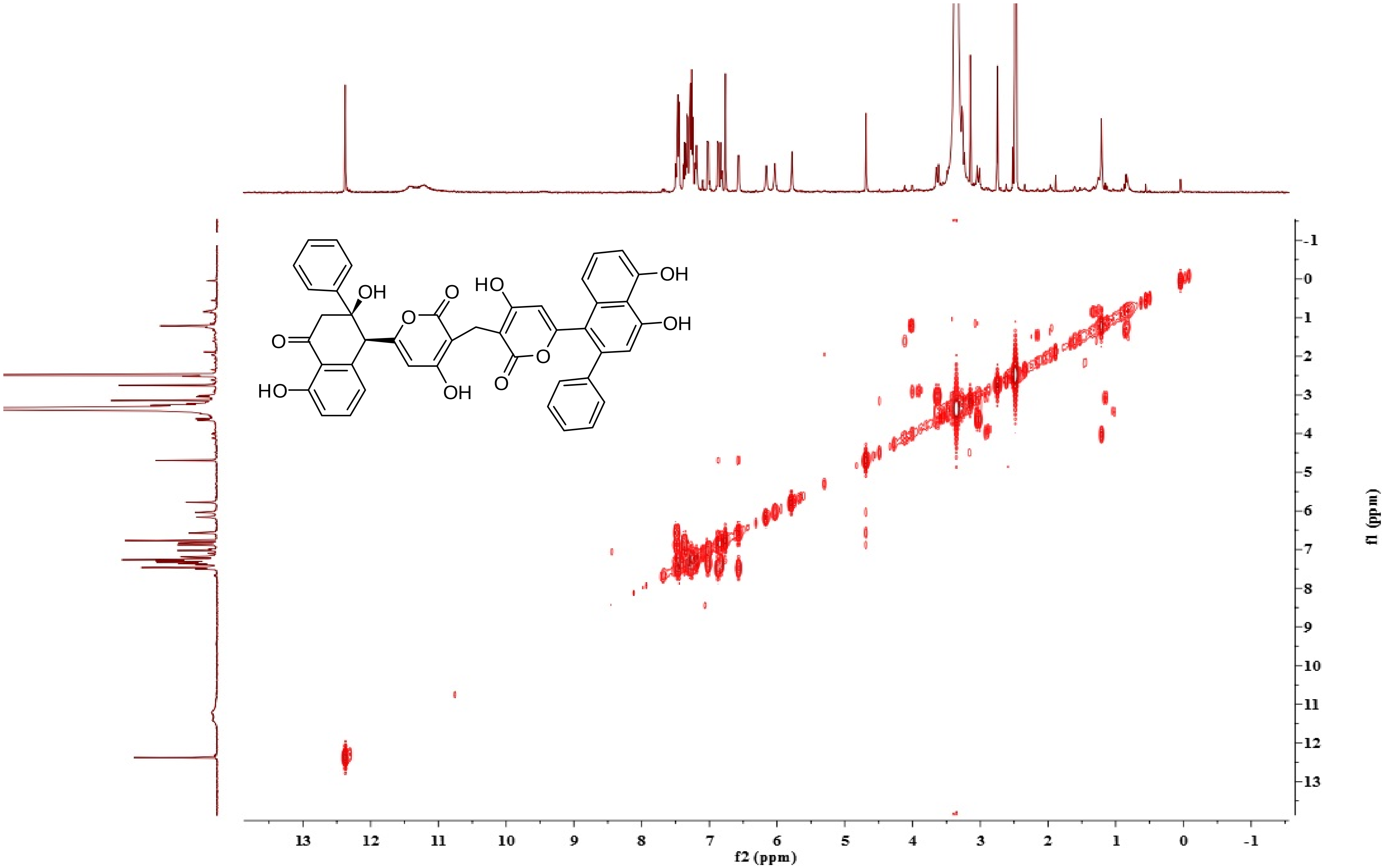

Figure S7. The enlarged ^{1}H-^{1}H COSY spectrum of wailupemycin H (1) in DMSO-d_6 (δ_H 6.40- δ_H 7.60)

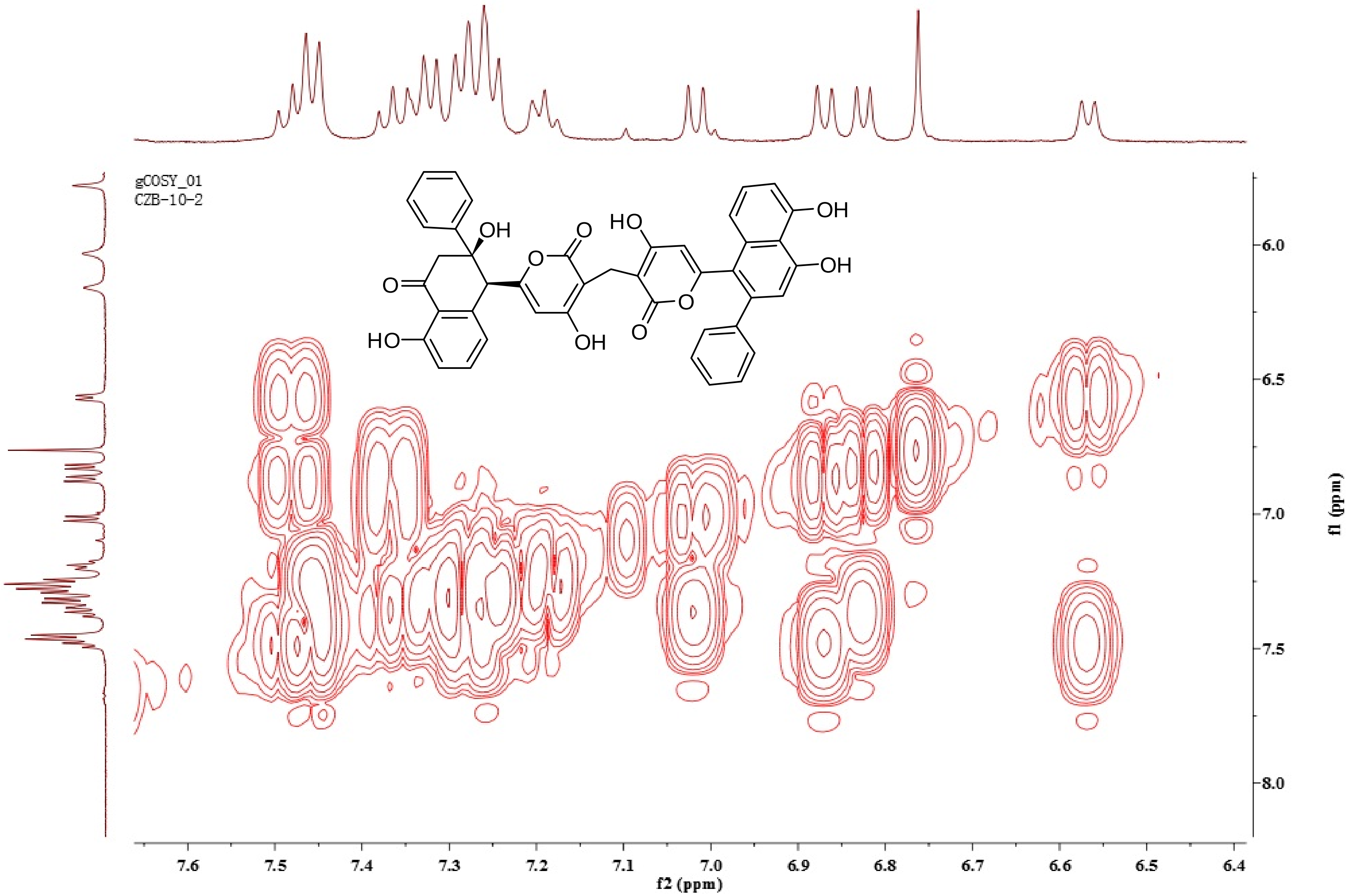

Figure S8. The HMBC spectrum of wailupemycin H (1) in DMSO-d_6

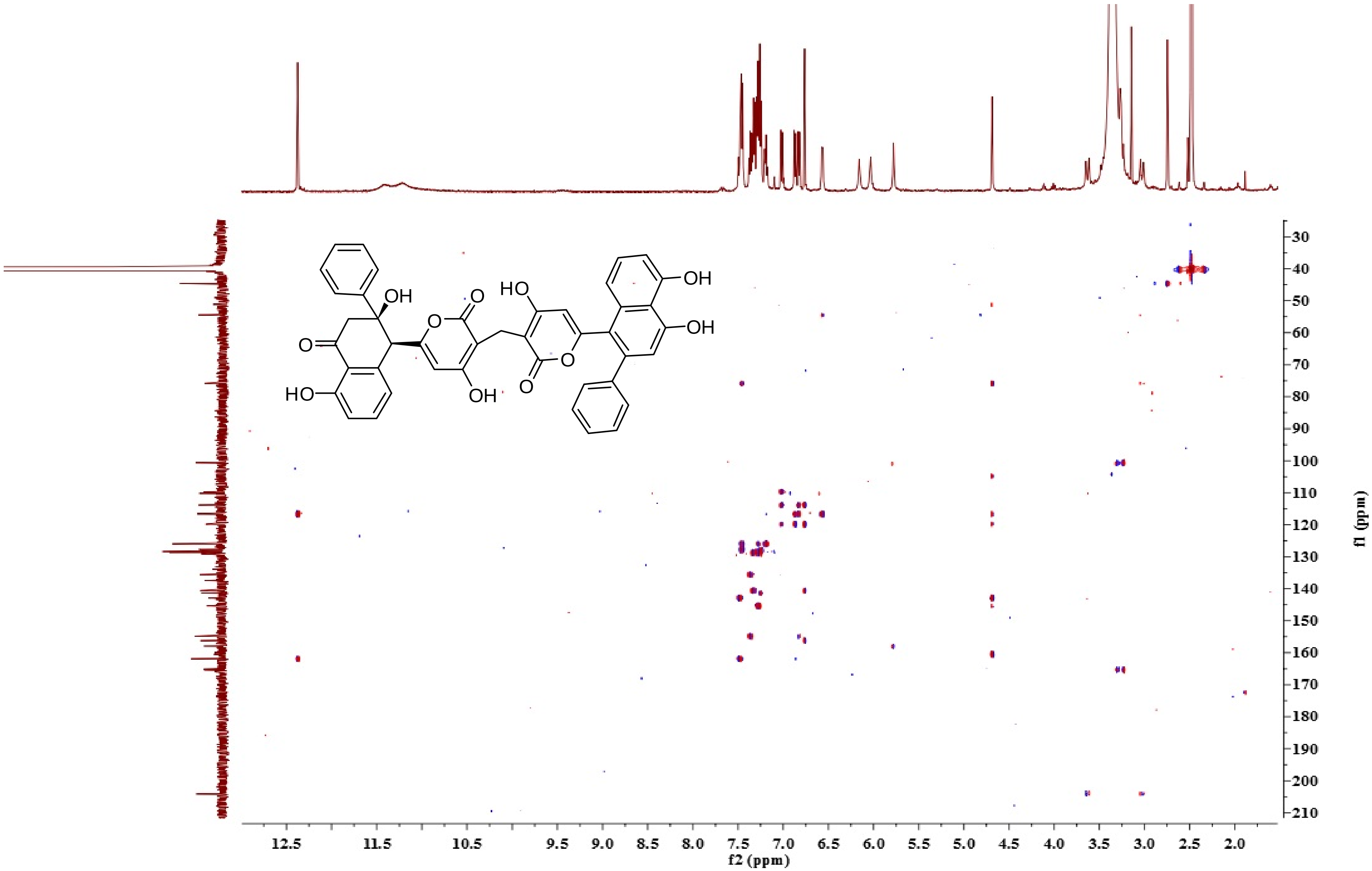

Figure S9. The enlarged HMBC spectrum of wailupemycin H (1) in DMSO-d_6 (δ_H 5.70- δ_H 7.60)

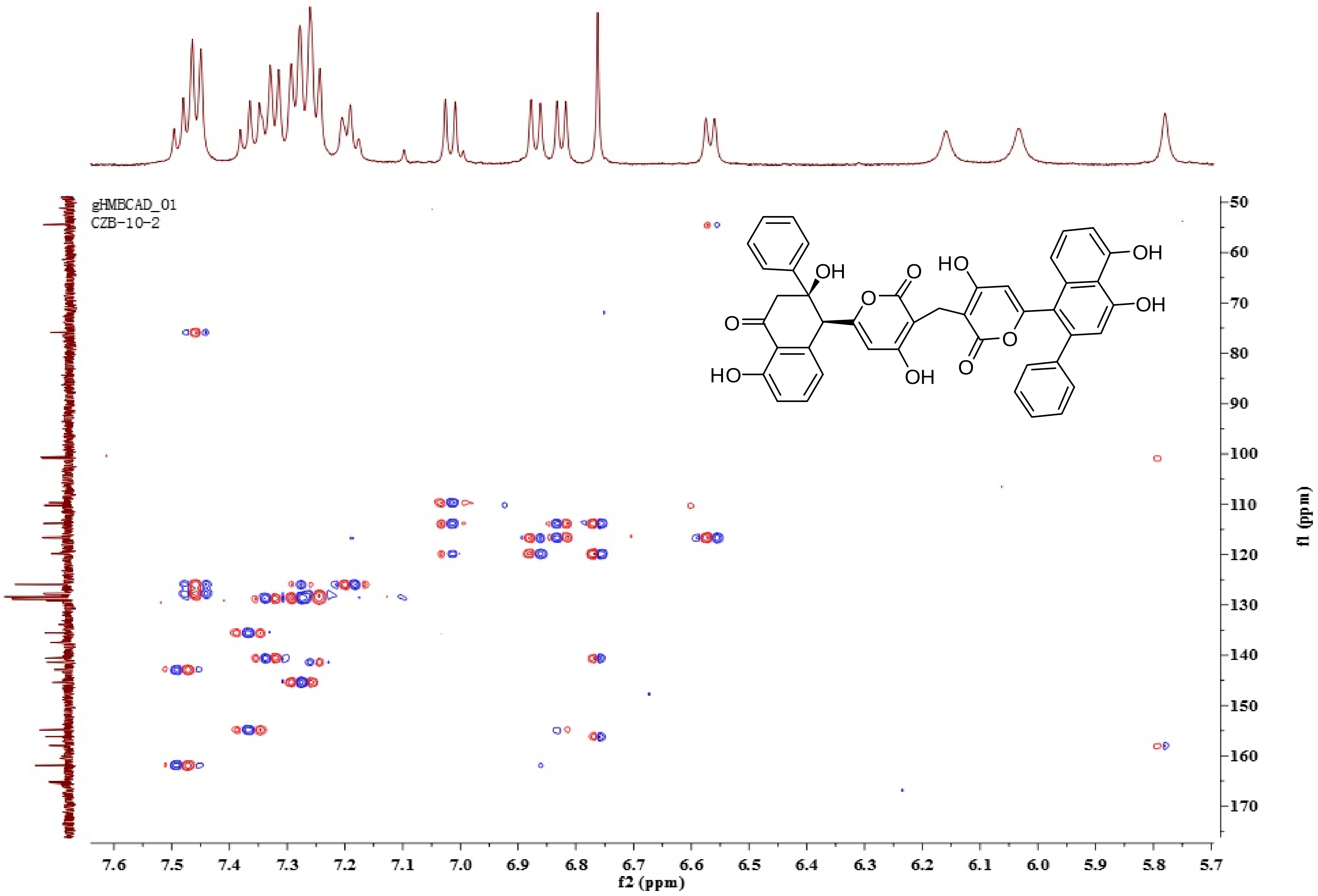

Figure S10. The enlarged HMBC spectrum of wailupemycin H (1) in DMSO-d_6 (δ_H 3.10- δ_H 4.80)

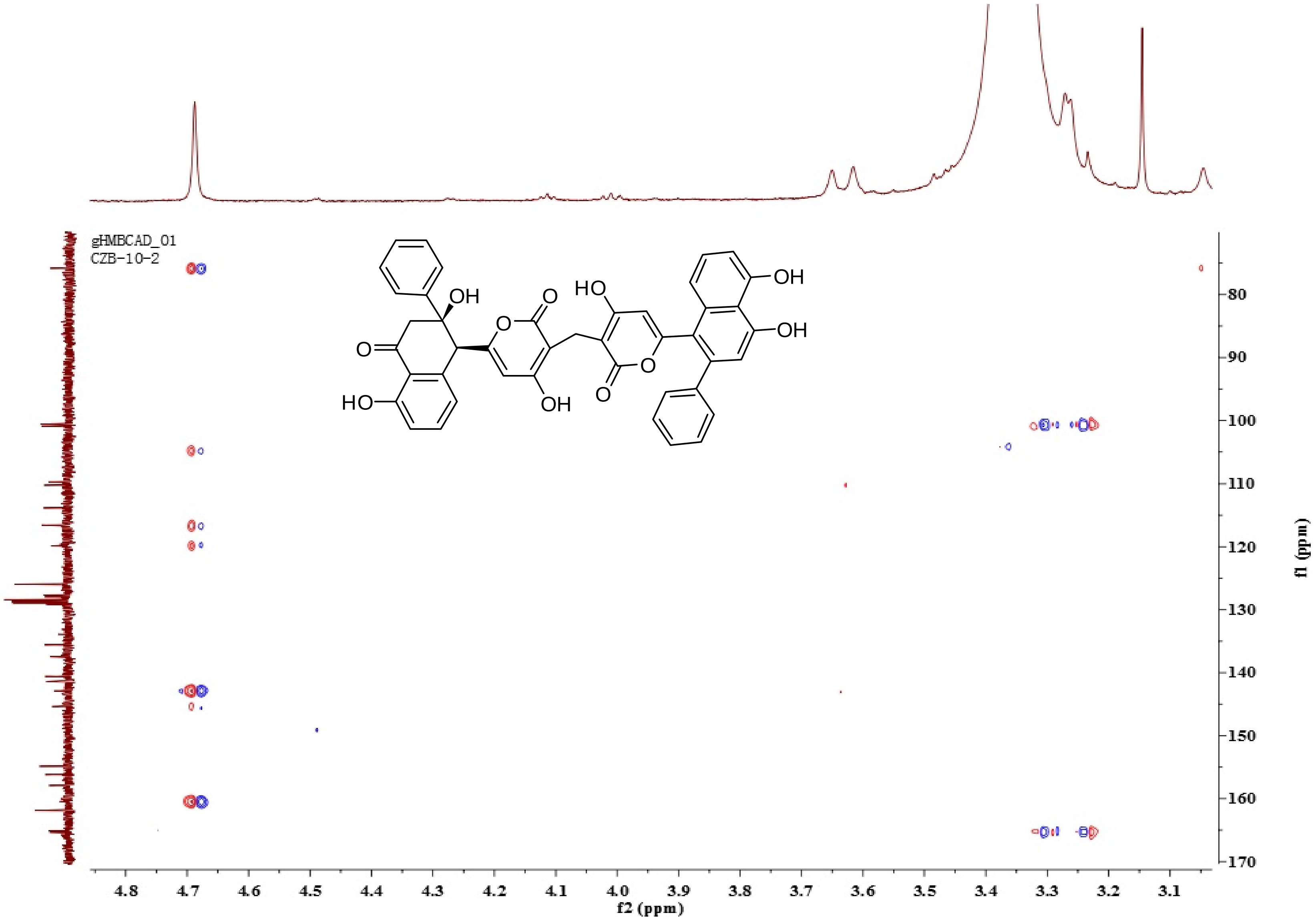

Figure S11. The NOSEY spectrum of wailupemycin H (1) in DMSO-d_6

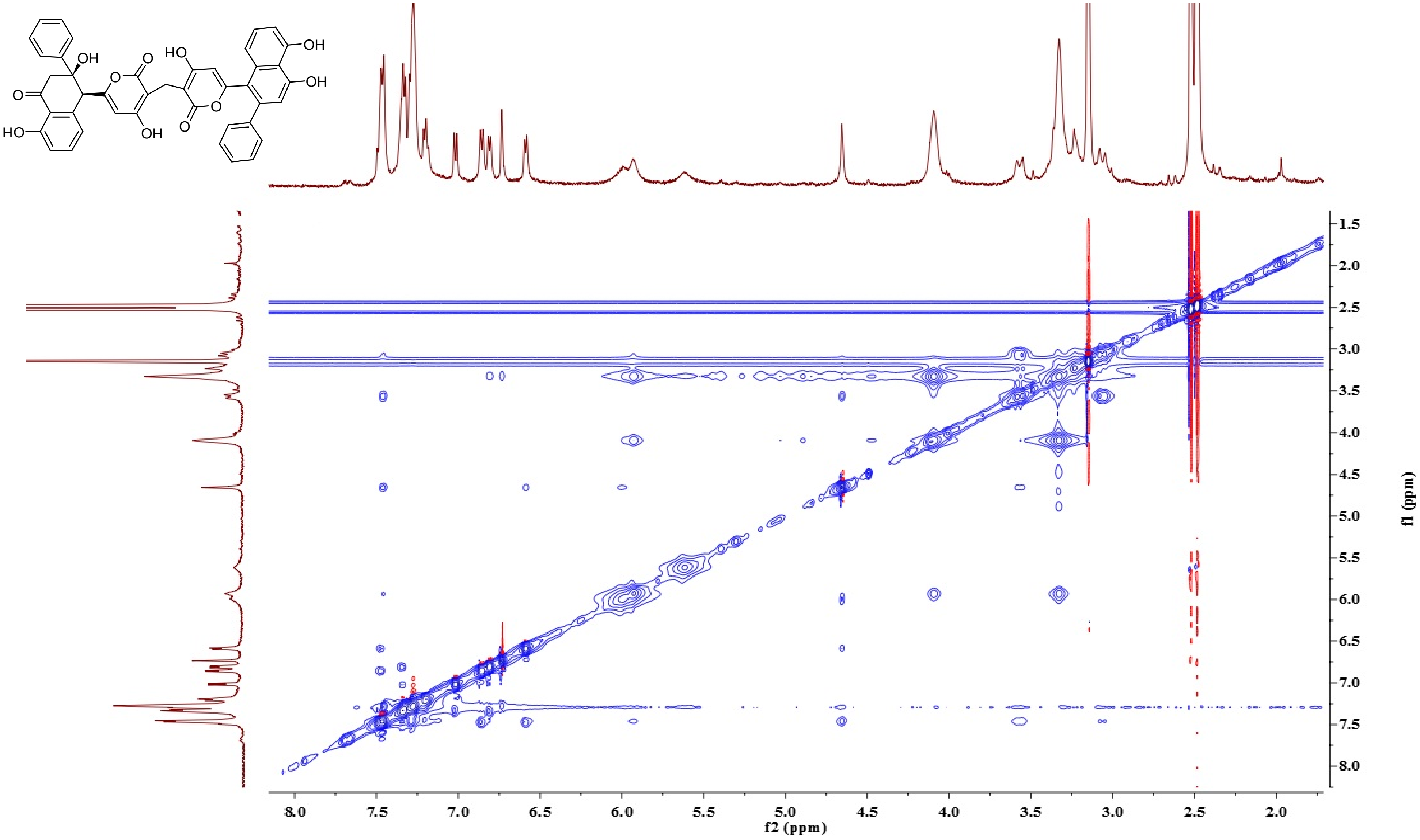

Figure S12. The ^{1}H-NMR spectrum of wailupemycin I (2) in DMSO-d_6

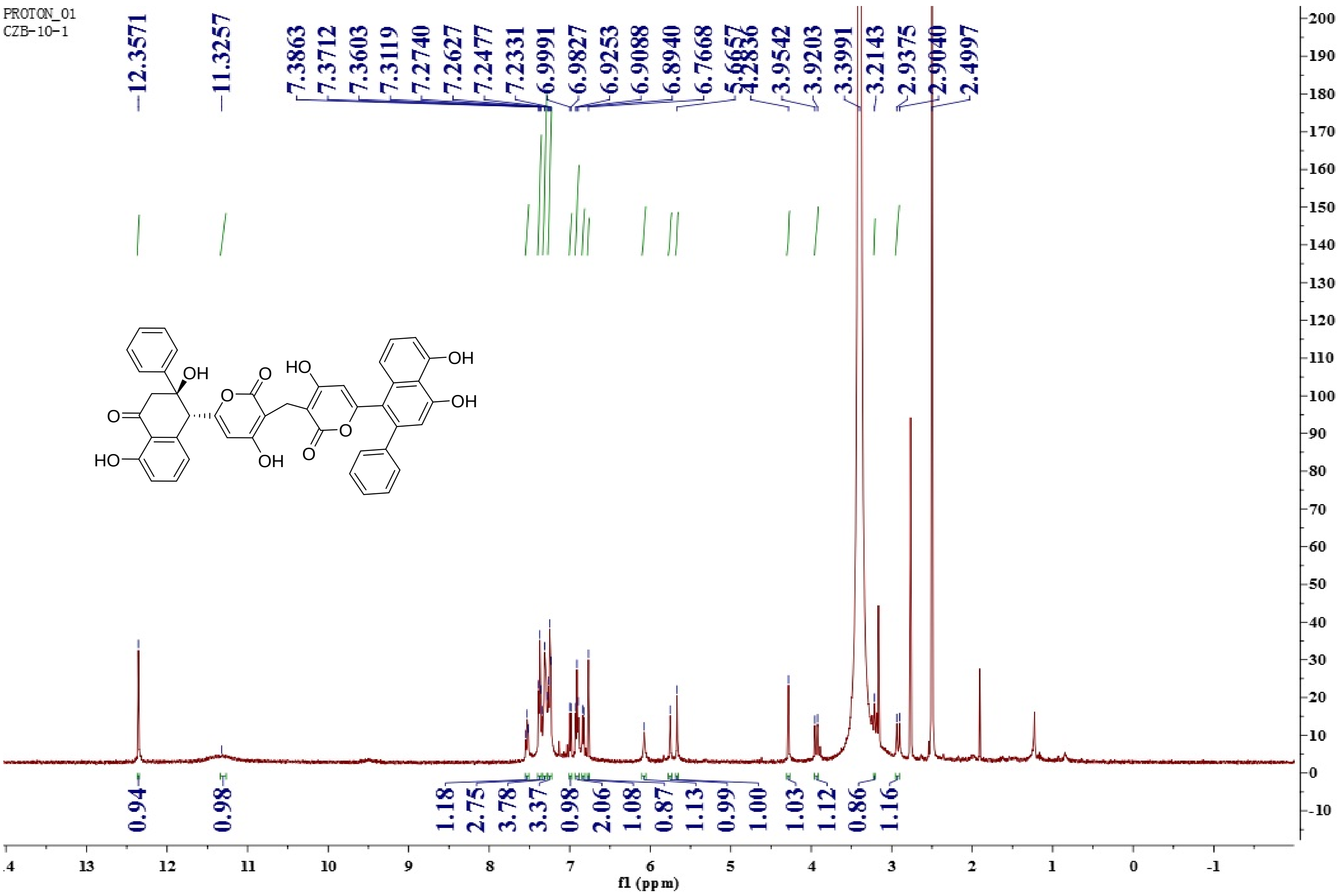

Figure S13. The ^{13}C-NMR spectrum of wailupemycin I (2) in DMSO-d_6

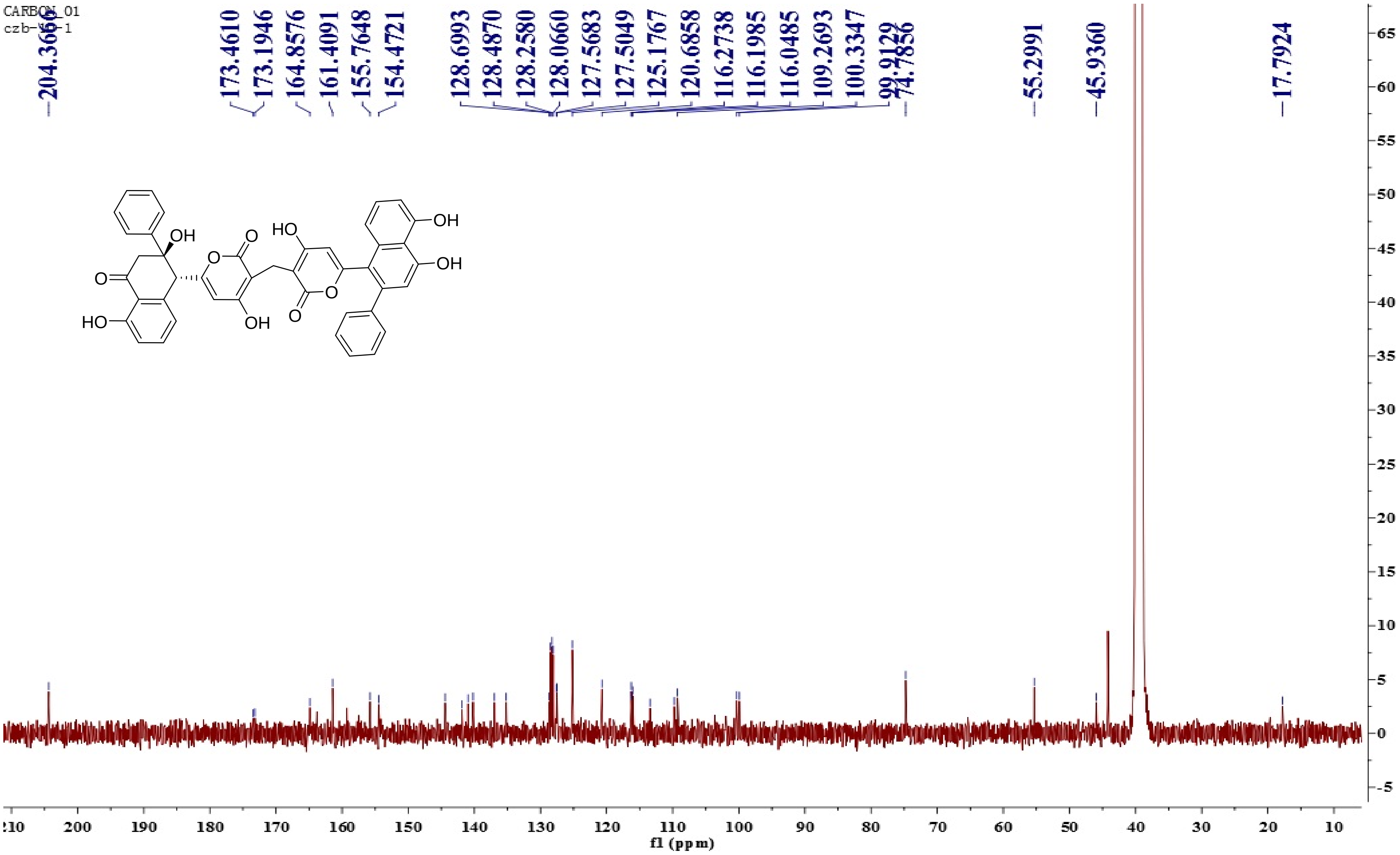

Figure S14. The DEPT spectrum of wailupemycin I (2) in DMSO-d_6

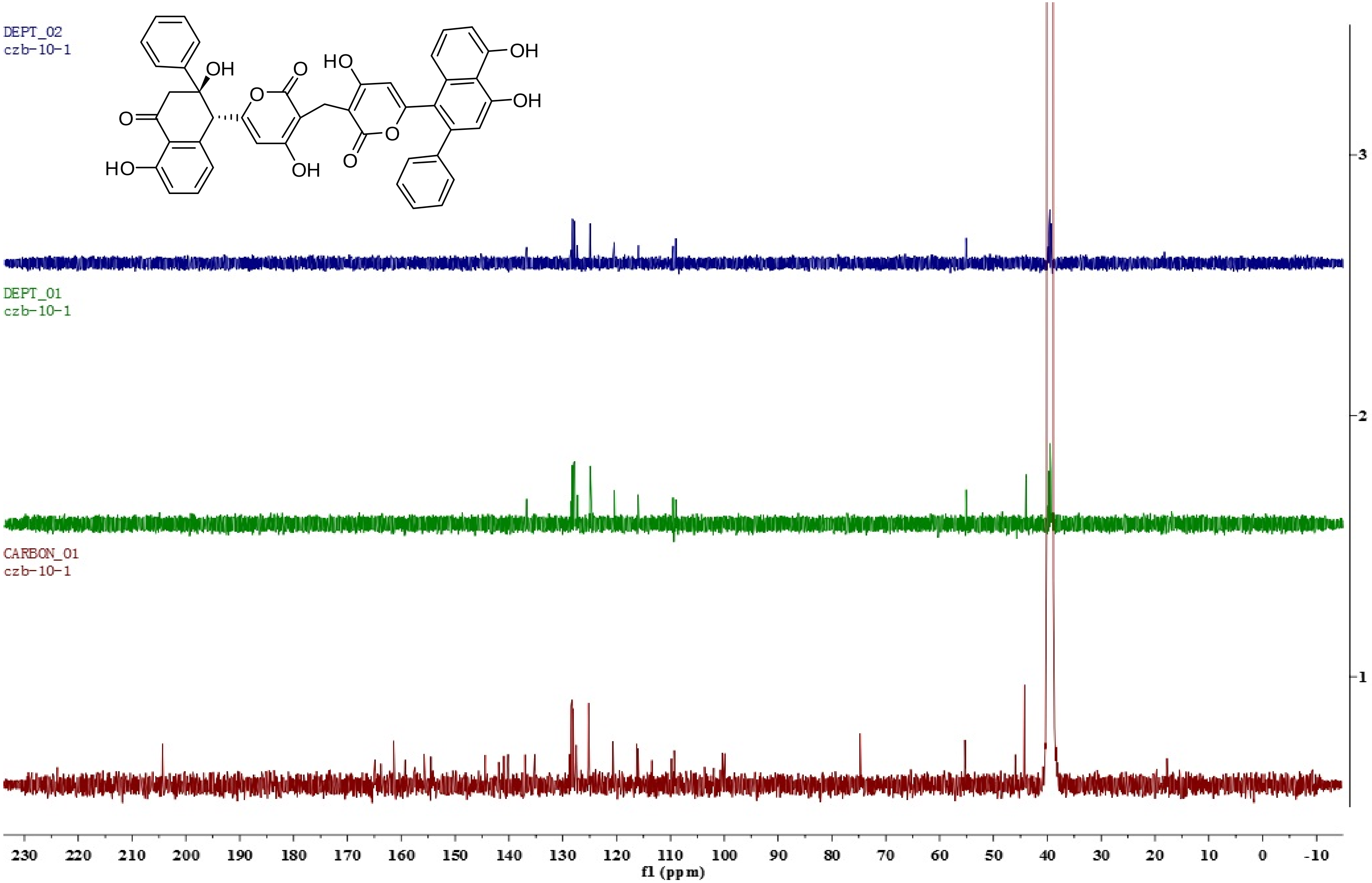

Figure S15. The HMQC spectrum of wailupemycin I (2) in DMSO-d_6

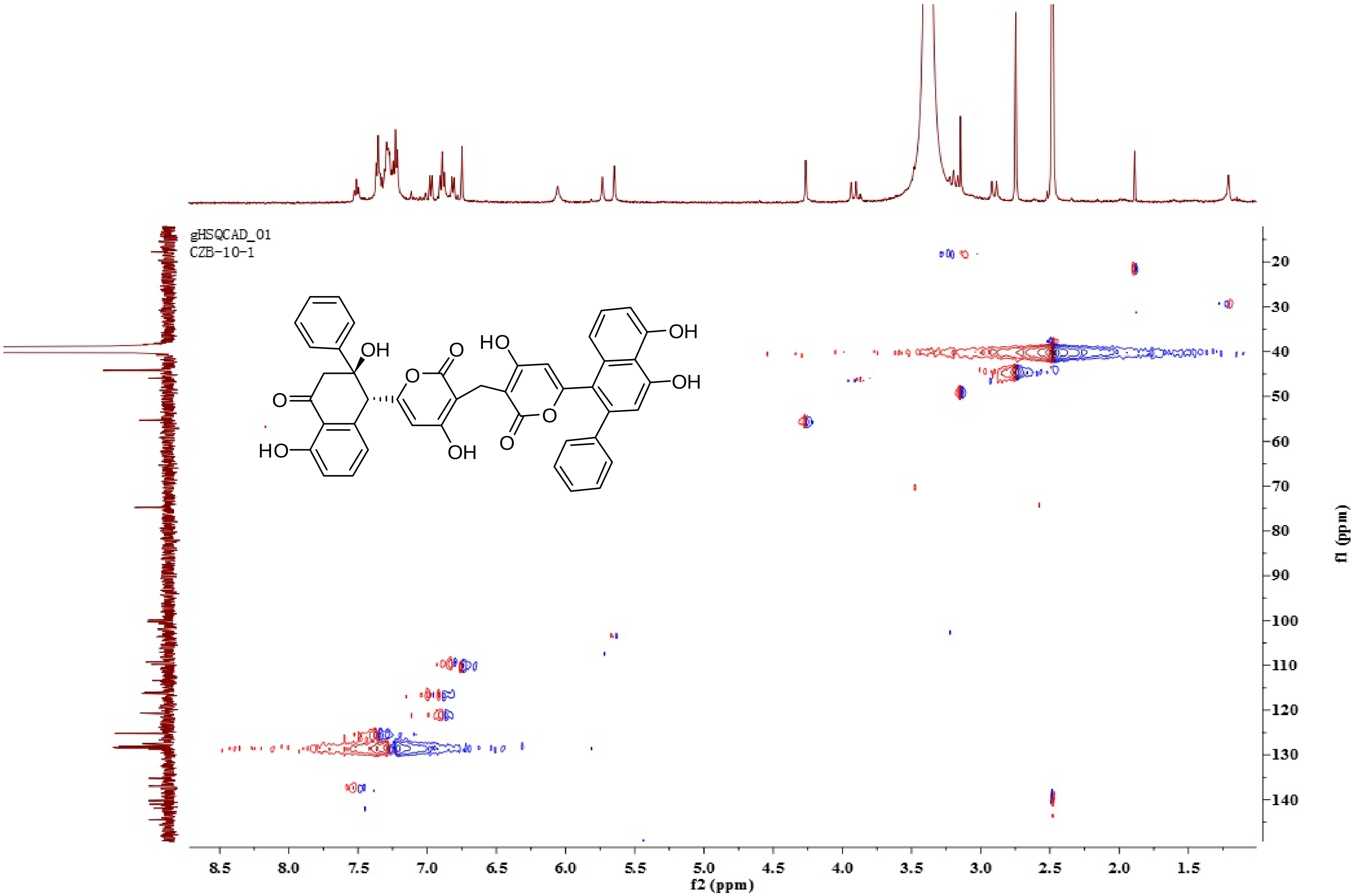

Figure S16. The enlarged HMQC spectrum of wailupemycin I (2) in DMSO-d_6 (δ_H 5.40- δ_H 7.90)

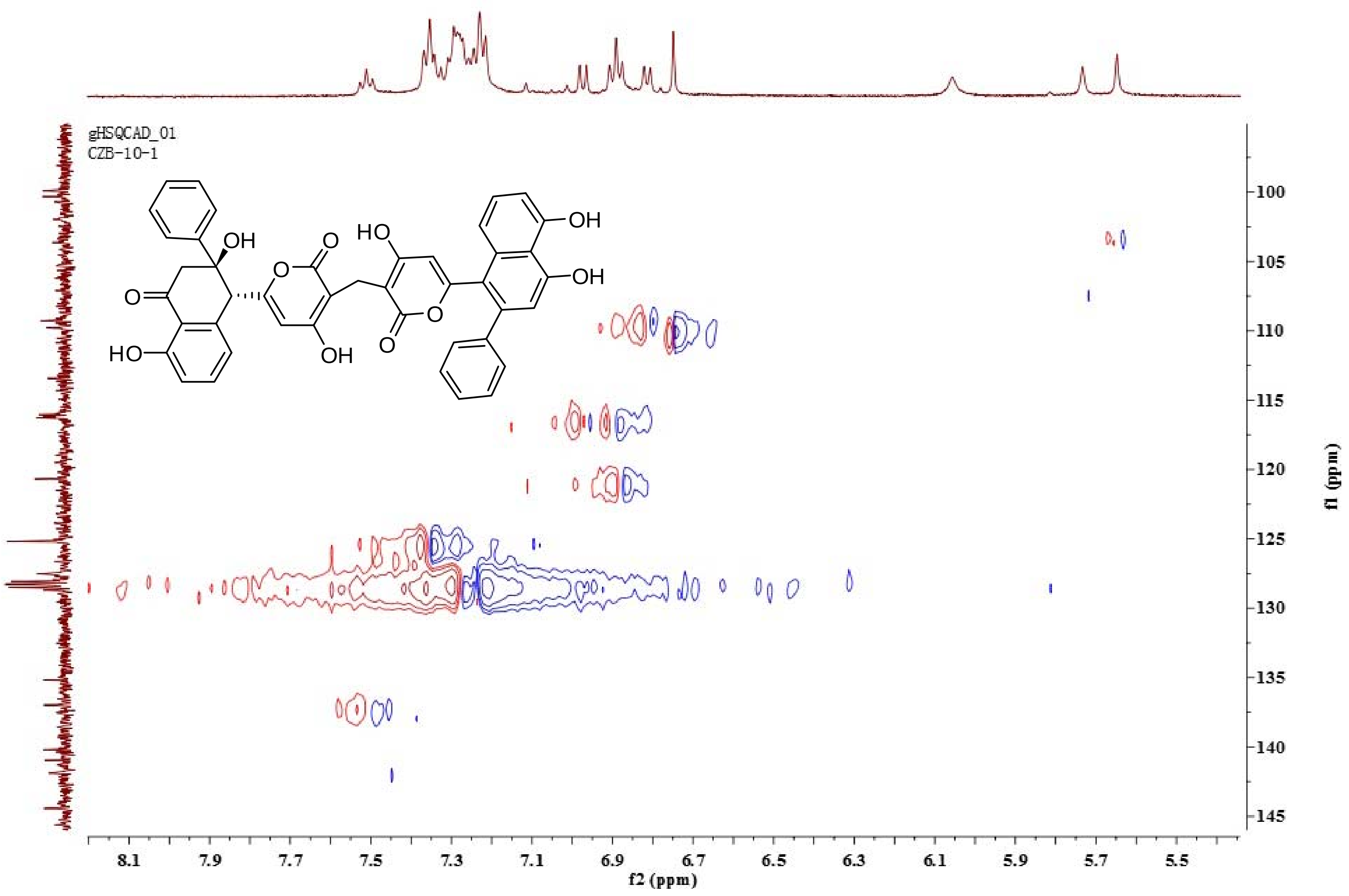

Figure S17. The ^{1}H-^{1}H COSY spectrum of wailupemycin I (2) in DMSO-d_6

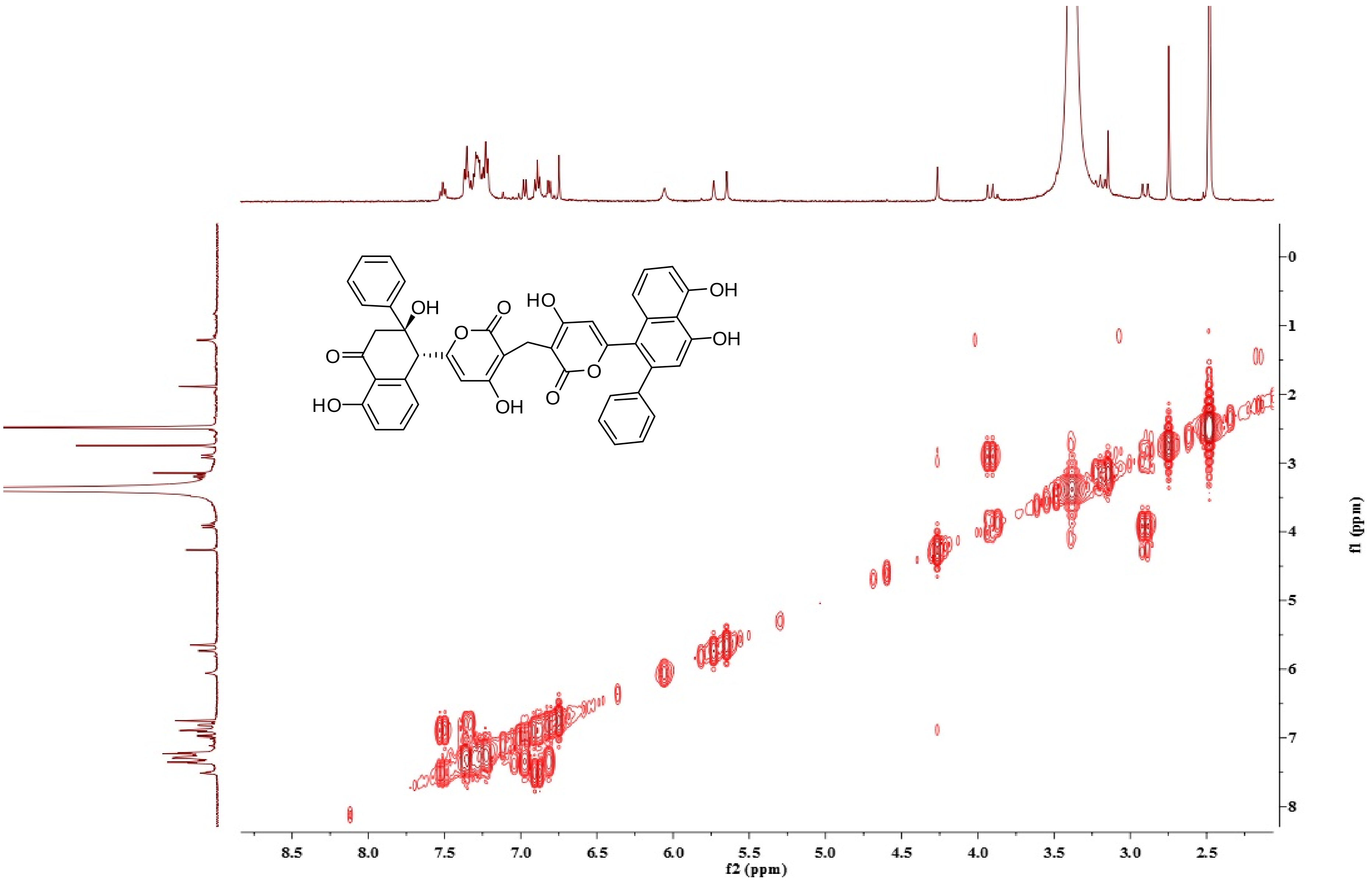

Figure S18. The enlarged ^{1}H-^{1}H COSY spectrum of wailupemycin I (2) in DMSO-d_6 (δ_H 6.65- δ_H 7.65)

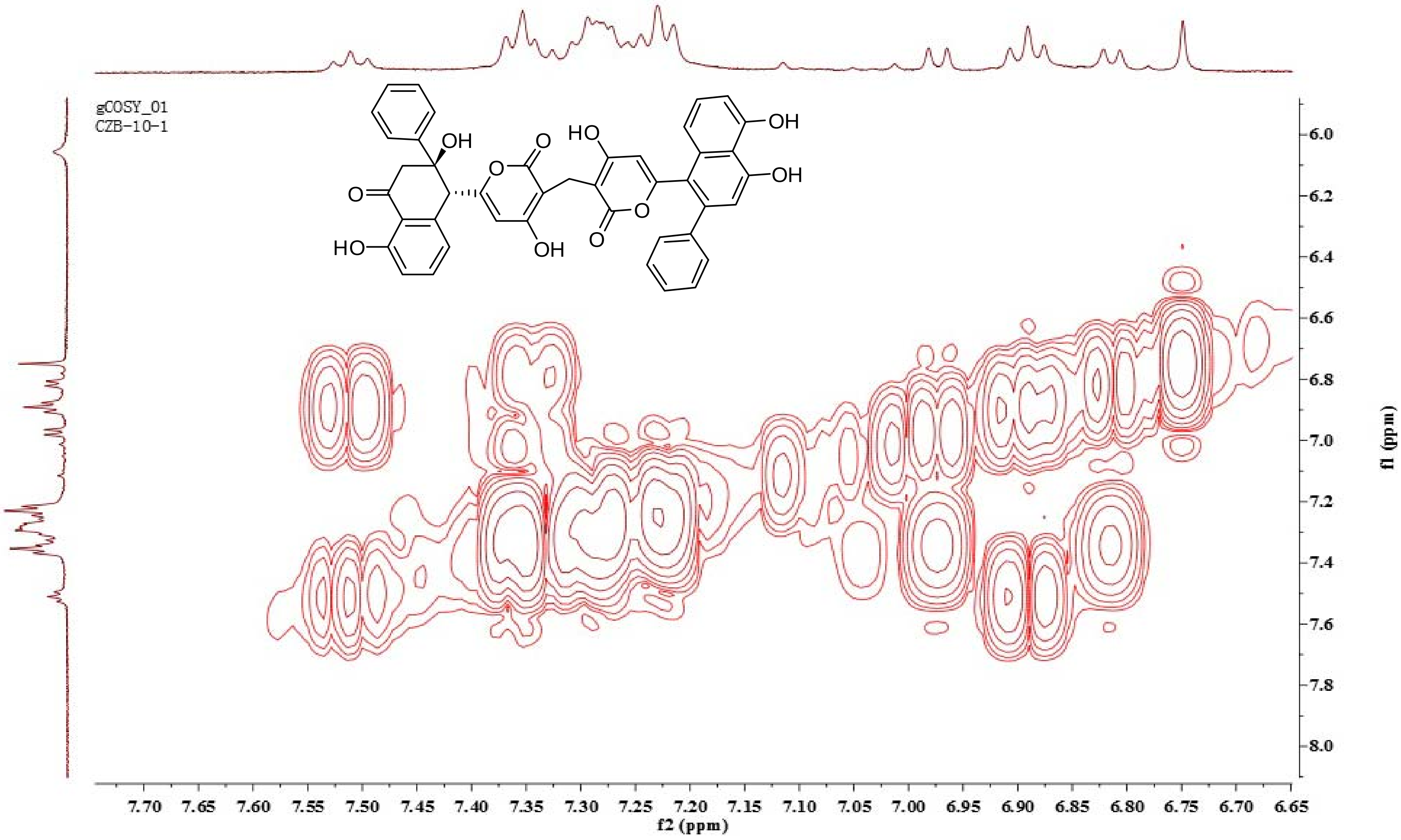

Figure S19. The HMBC spectrum of wailupemycin I (2) in DMSO-d_6

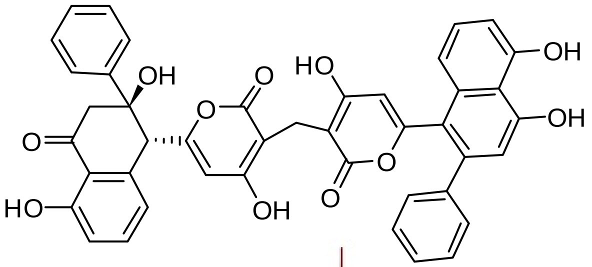

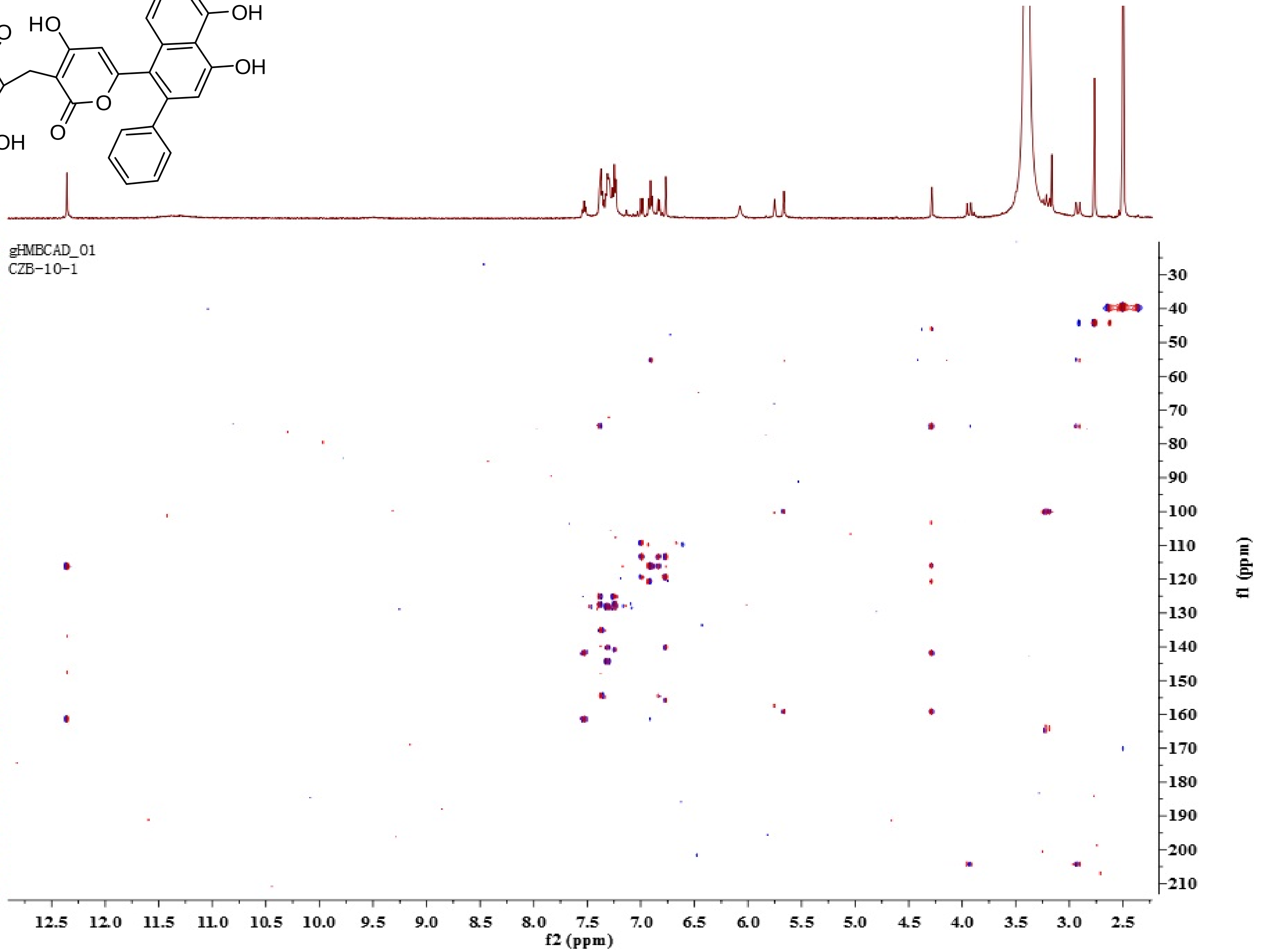

Figure S20. The enlarged HMBC spectrum of wailupemycin I (2) in DMSO-d_6 (δ_H 5.40- δ_H 7.70)

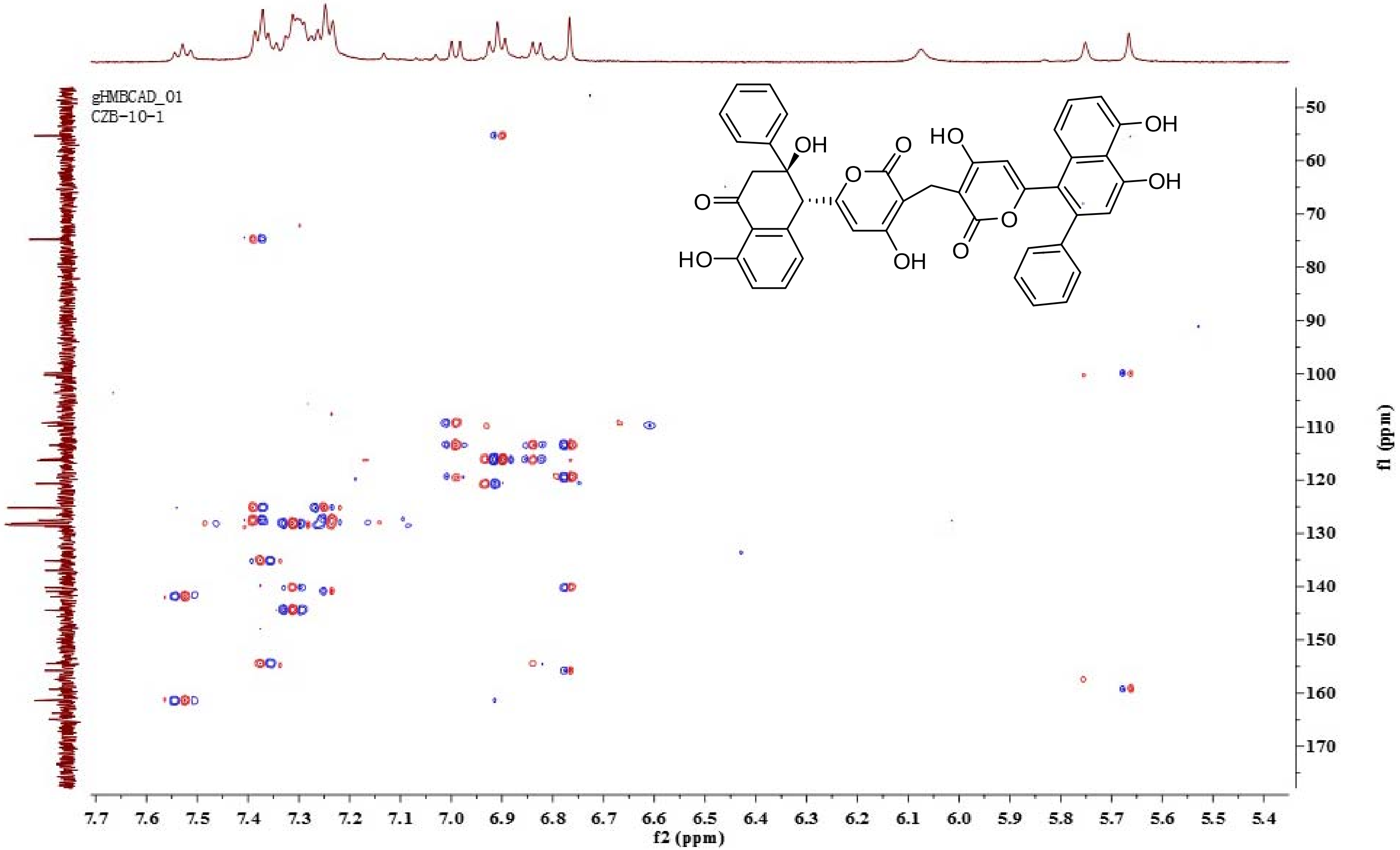

Figure S21. The enlarged HMBC spectrum of wailupemycin I (2) in DMSO-d_6 (δ_H 3.10- δ_H 4.60)

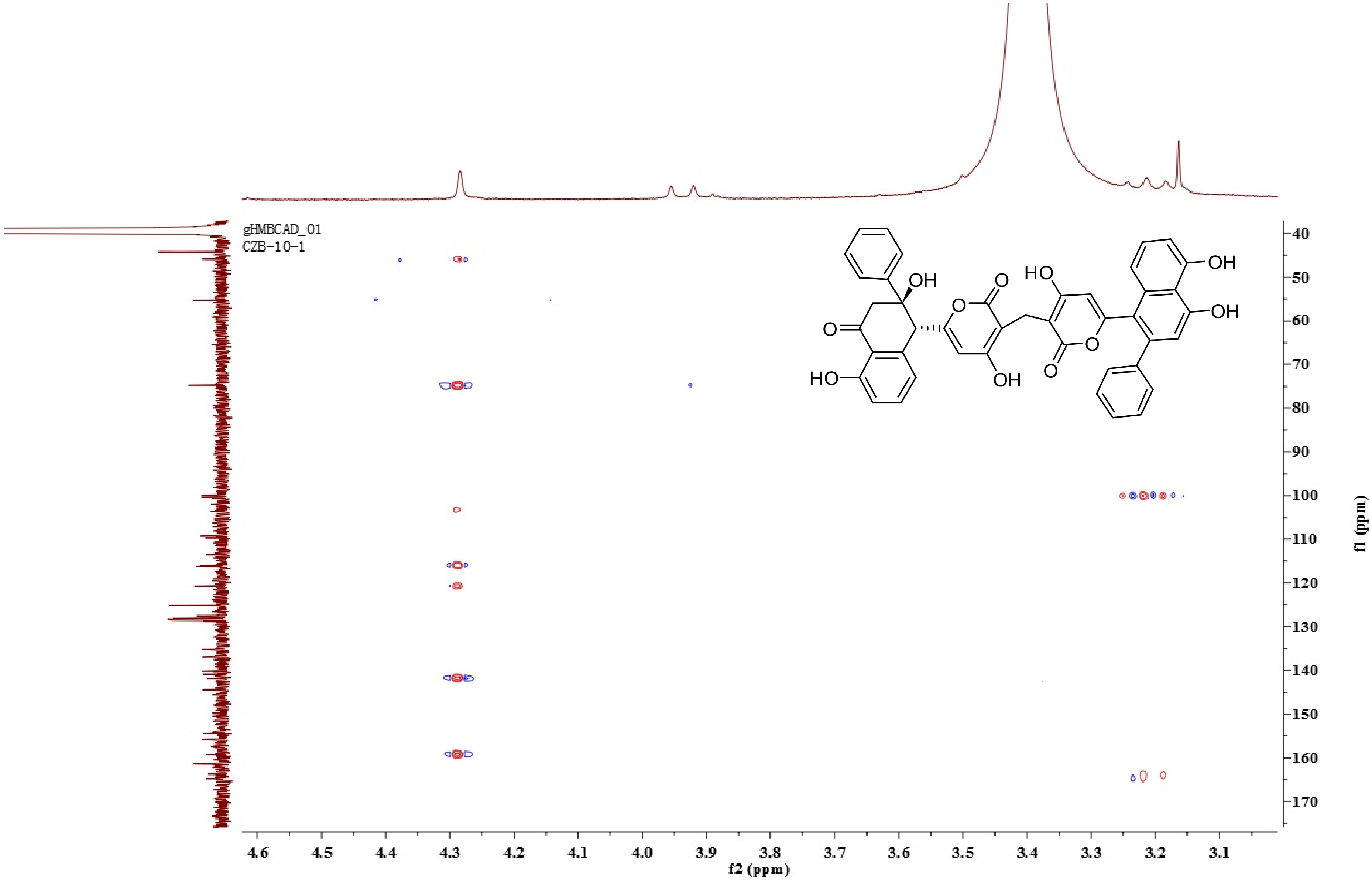

Figure S22. The NOSEY spectrum of wailupemycin I (2) in DMSO-d_6

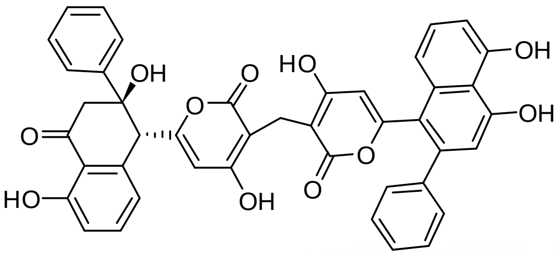

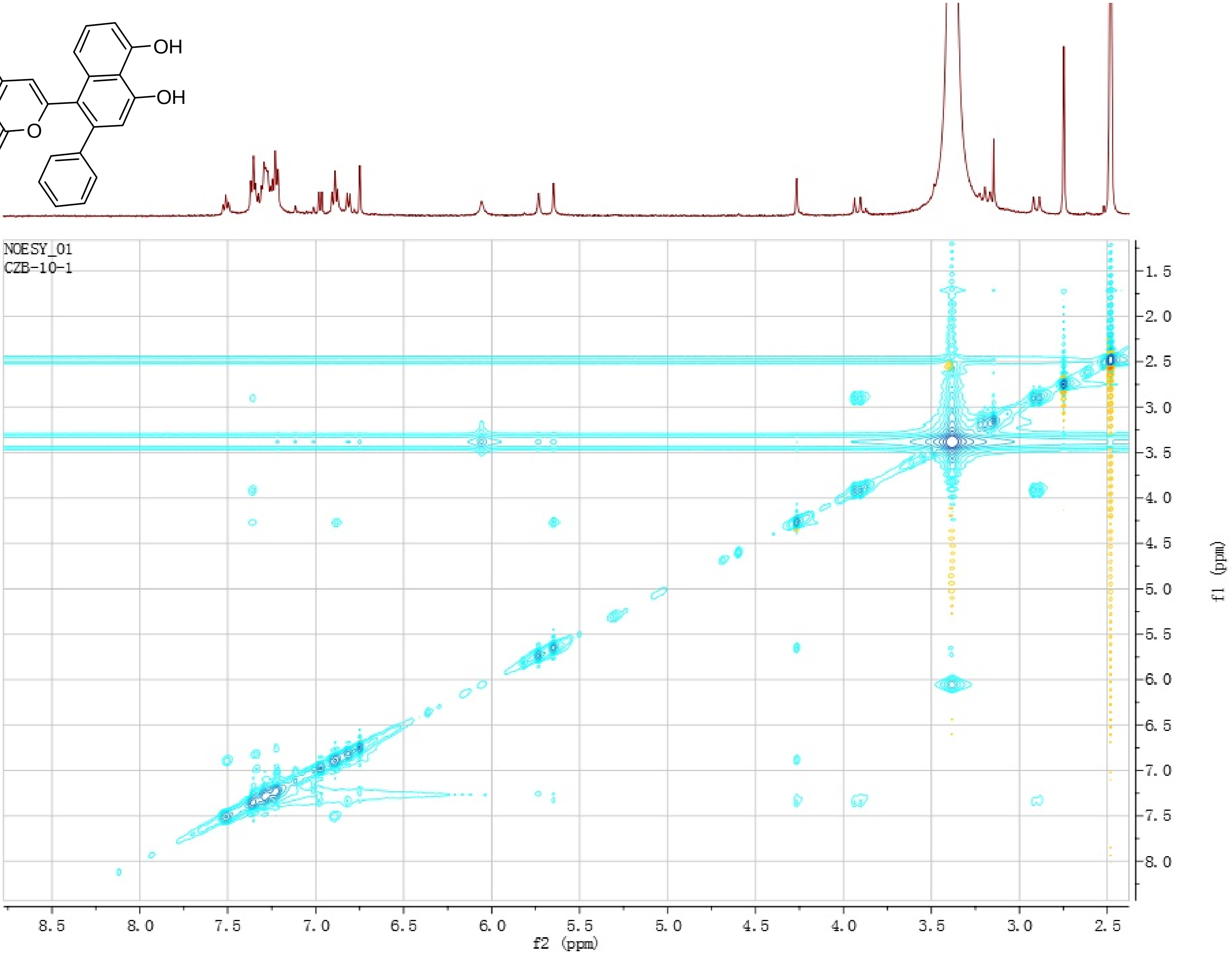

Figure S23. The ^{1}H-NMR spectrum of wailupemycin D (3) in DMSO-d_6

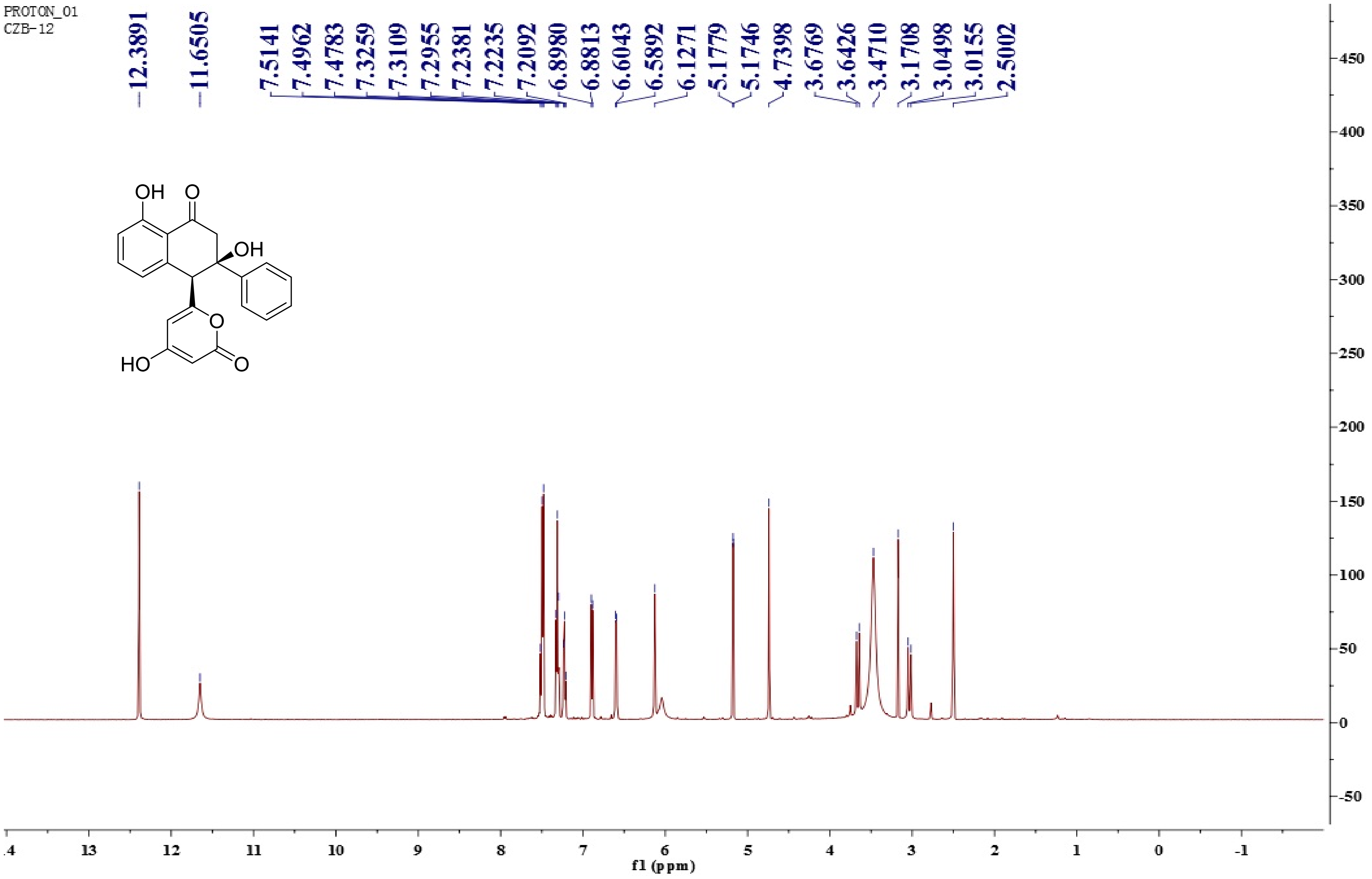

Figure S24. The ^{13}C-NMR spectrum of wailupemycin D (3) in DMSO-d_6

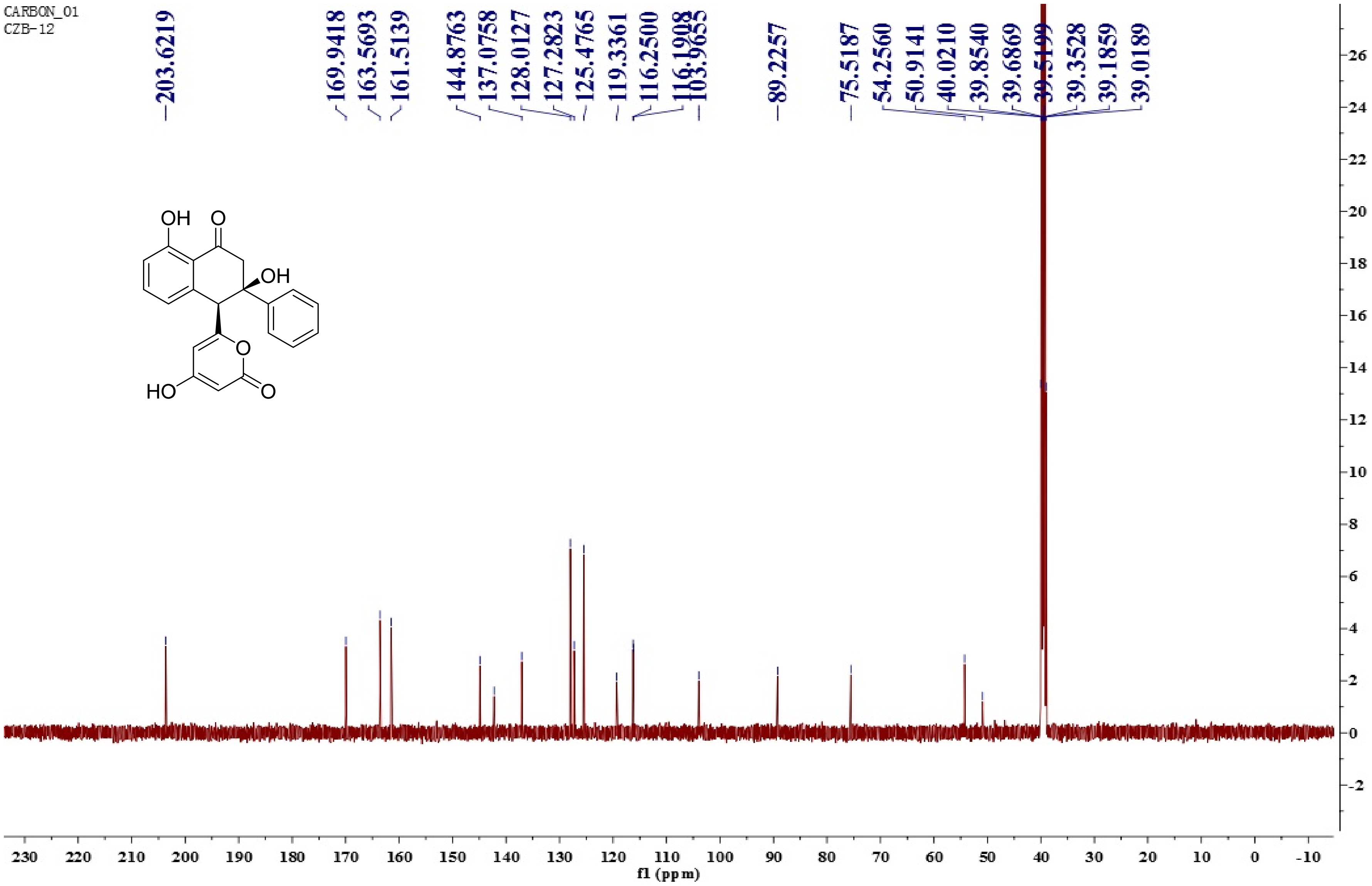

Figure S25. The DEPT spectrum of wailupemycin D (3) in DMSO-d_6

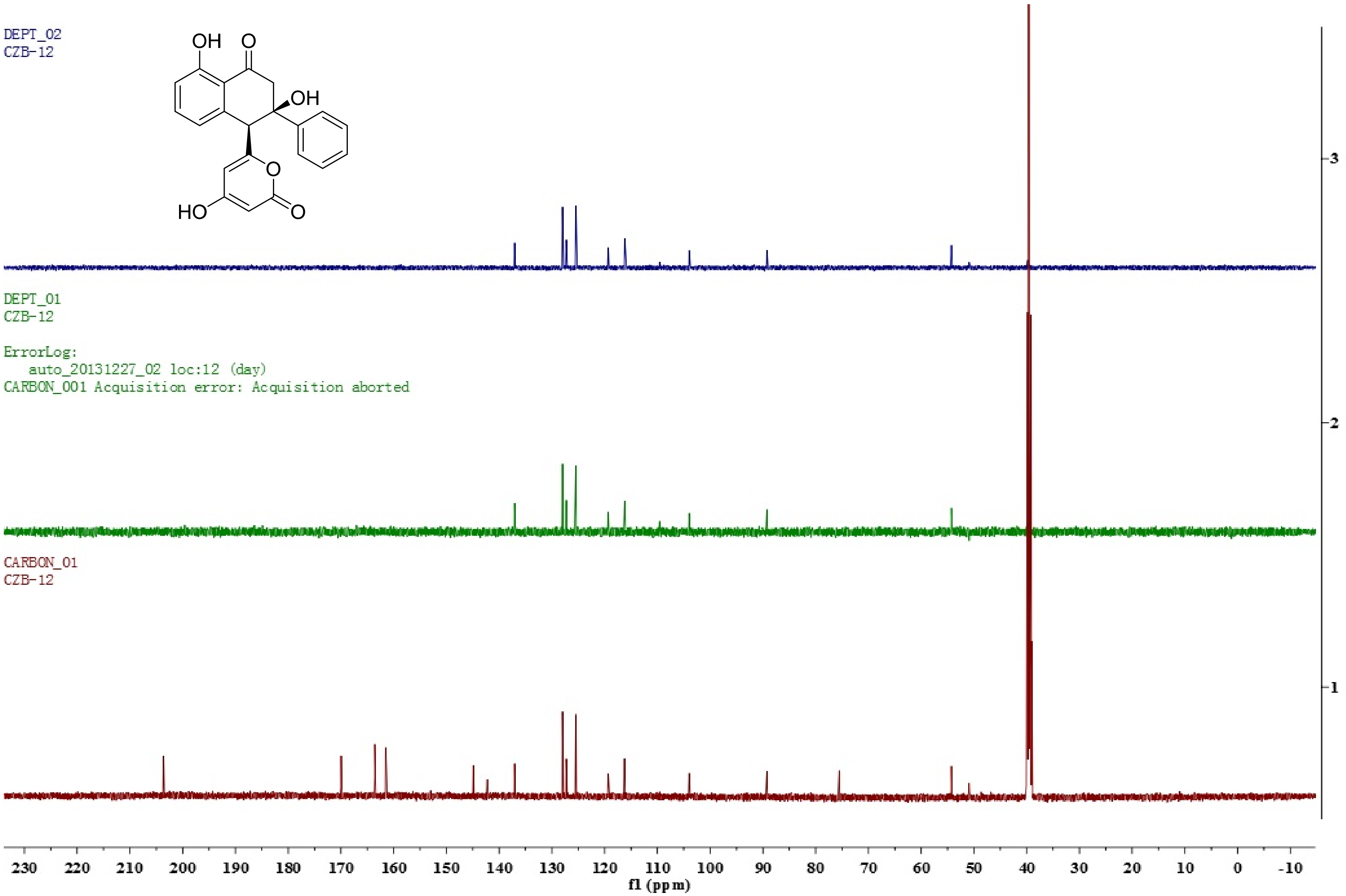

Figure S26. The NOSEY spectrum of wailupemycin D (3) in DMSO-d_6

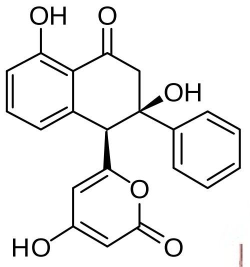

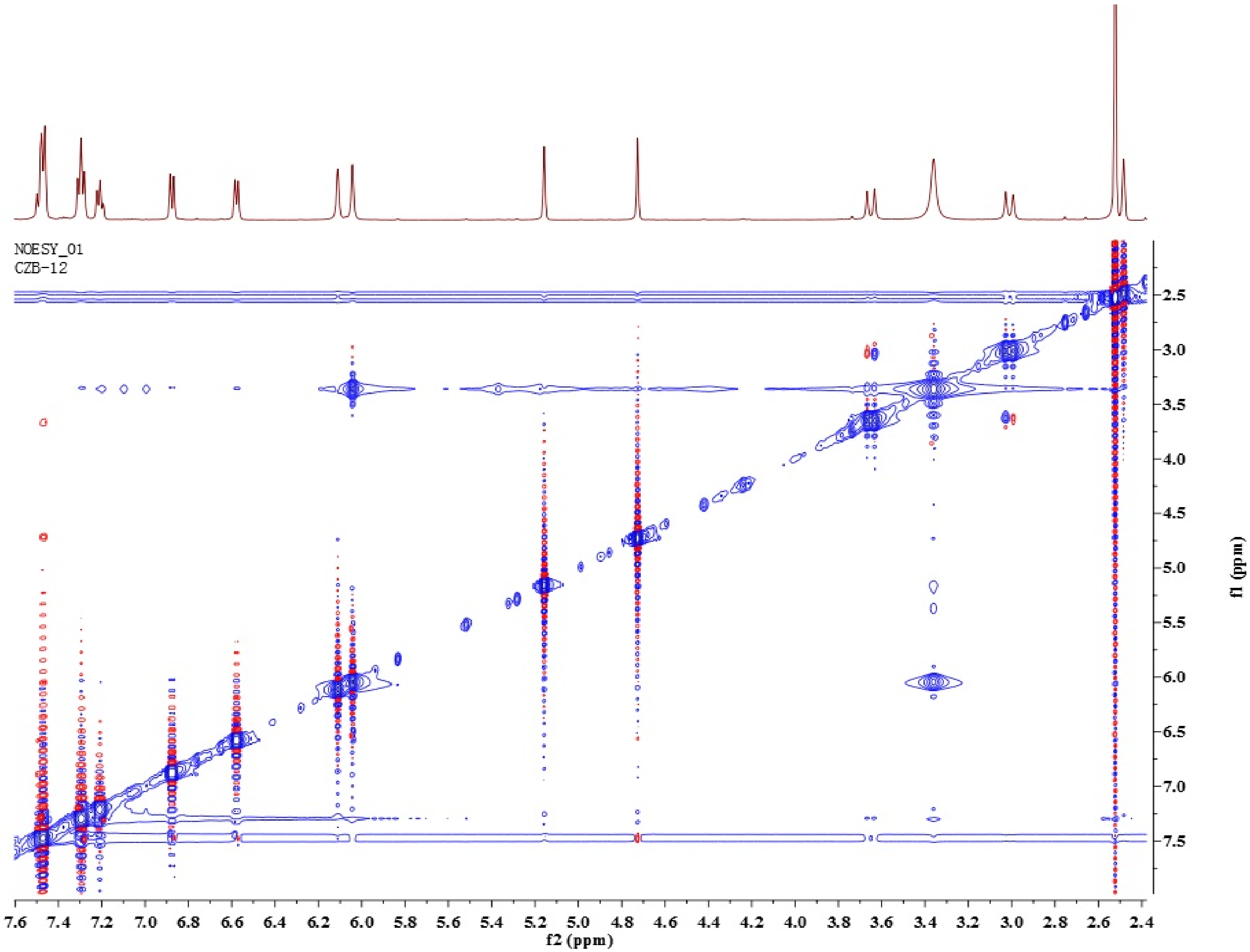

Figure S27. The ^{1}H-NMR spectrum of wailupemycin E (4) in DMSO-d_6

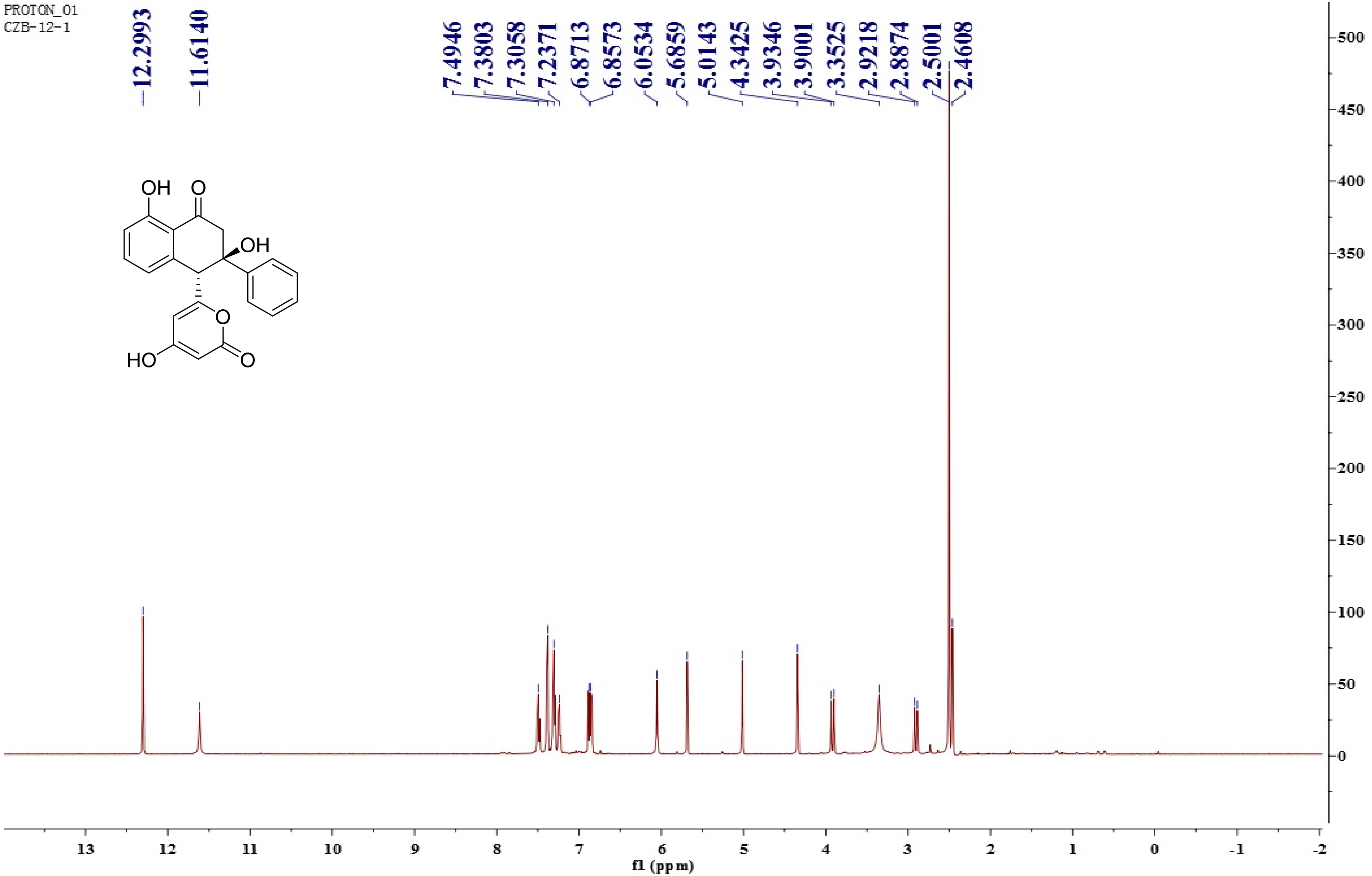

Figure S28. The ^{13}C-NMR spectrum of wailupemycin E (4) in DMSO-d_6

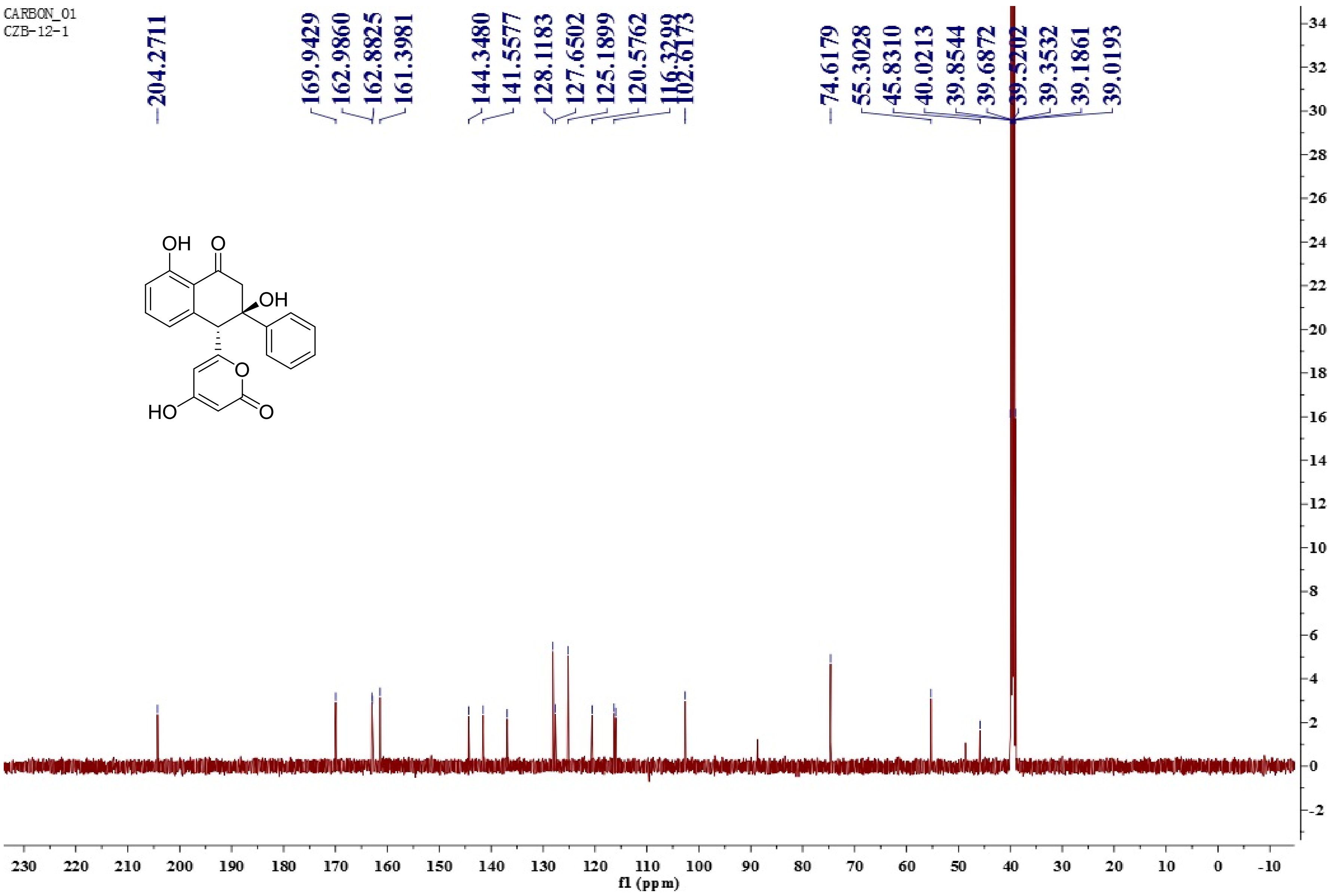

Figure S29. The DEPT spectrum of wailupemycin E (4) in DMSO-d_6

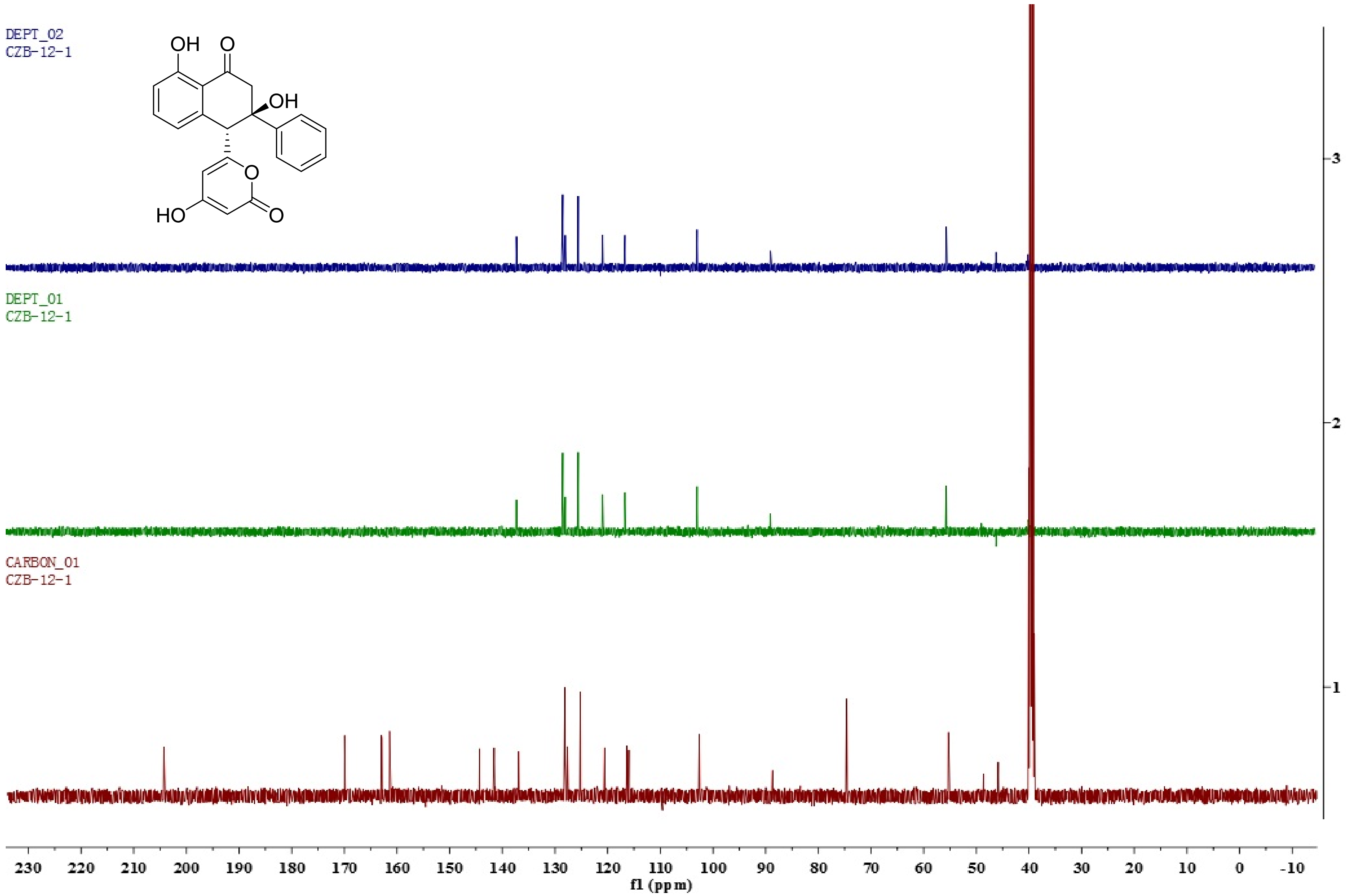

Figure S30. The NOSEY spectrum of wailupemycin E (4) in DMSO-d_6

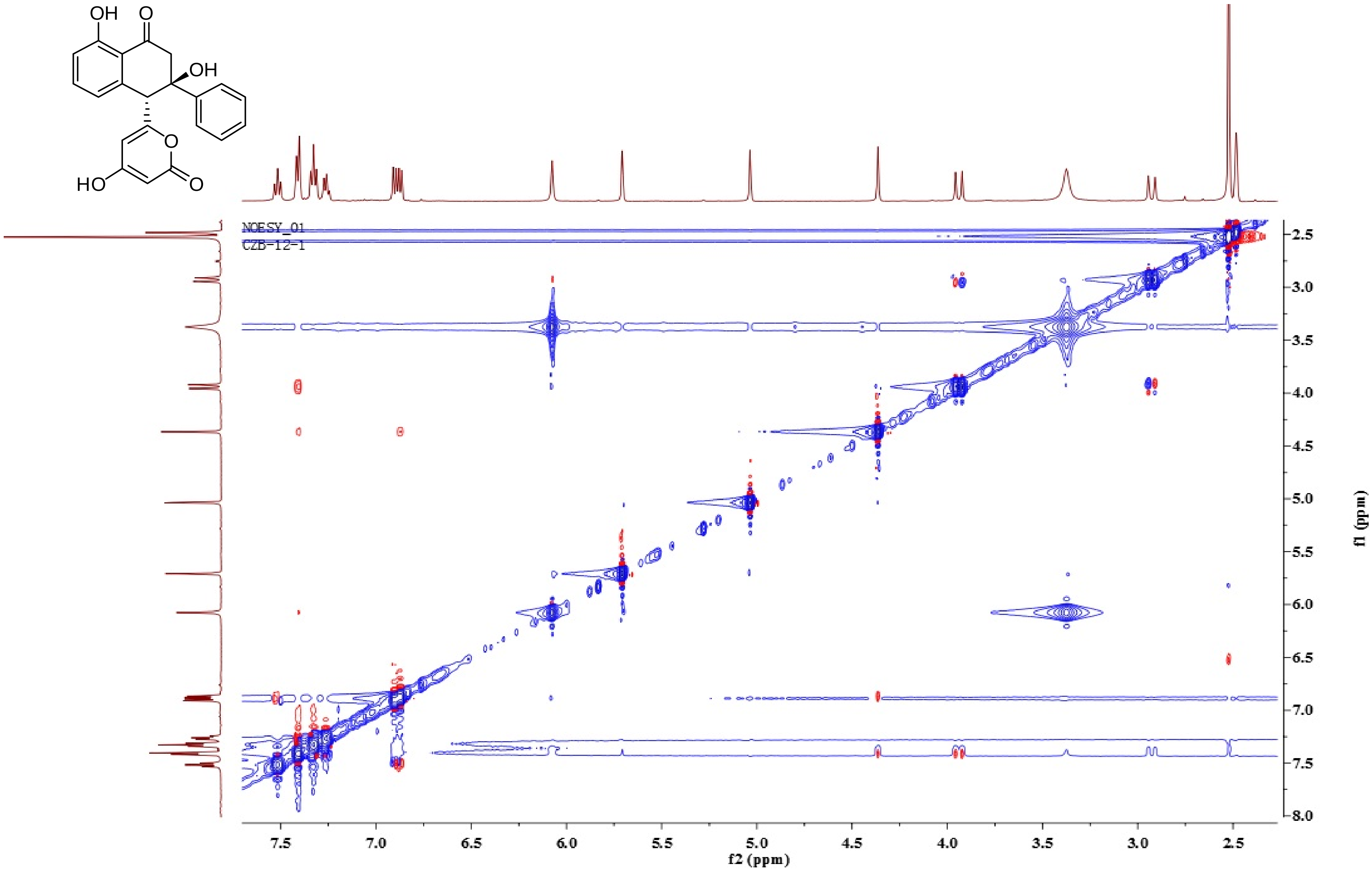

Figure S31. HPLC profiles of the products (A, B; solvent: 0-5 min, 5% MeCN, 5-20 min, 100% MeCN, 20-25min, 5% MeCN; flow rate: 1 mL/min; temperature: 30 °C; UV detection at λ 335 nm; Cholester packed column) and the identification of synthetic **1** and **2** by ESI-MS and co-HPLC experiments (solvent: 80% MeCN-H_2O; temperature: 30 °C; flow rate: 1 mL/min; UV detection at λ 335 nm; Cholester packed column) and.

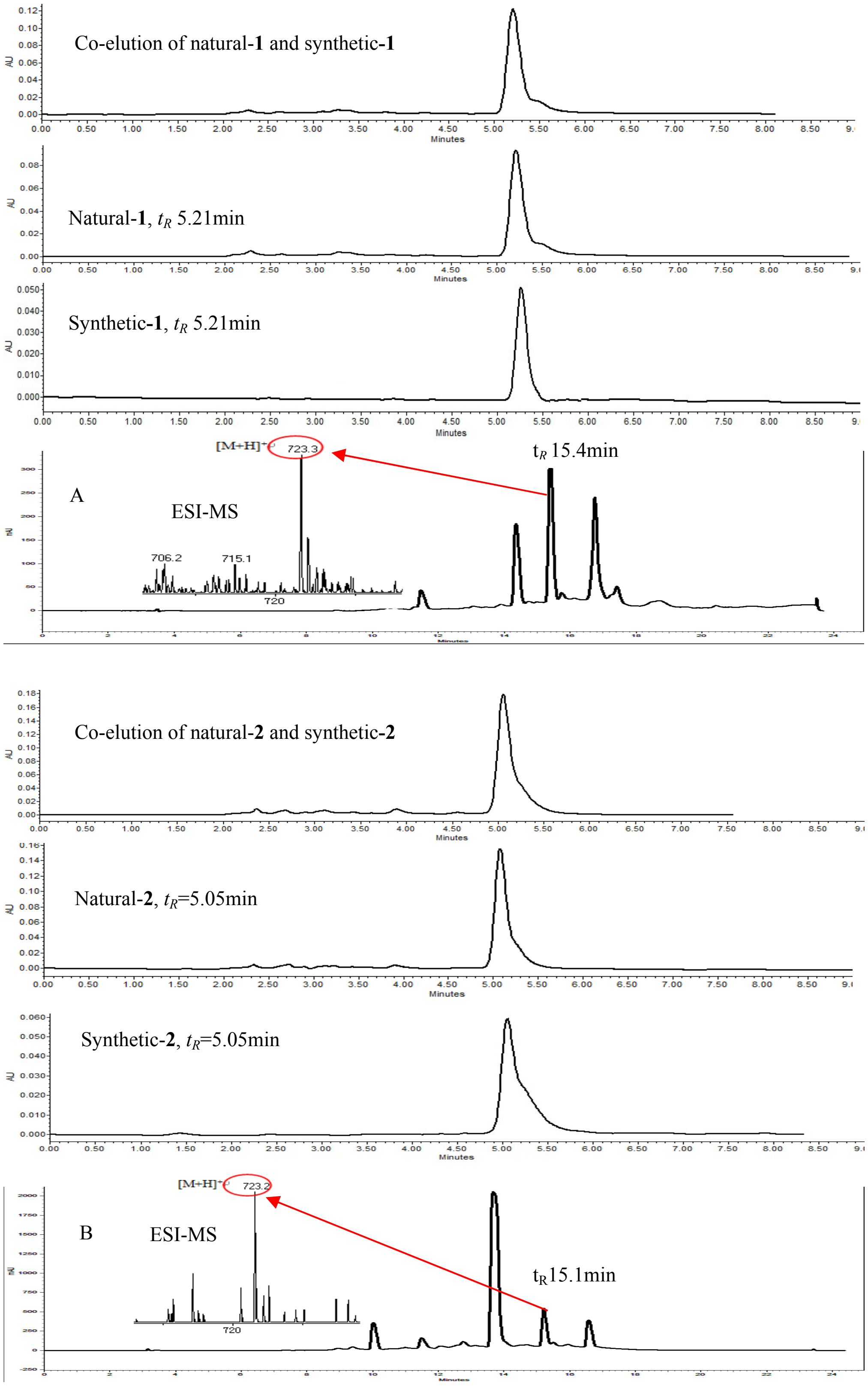

Table S2. Energy analysis for stable conformers of **3a** and **3b**

No.	Energy (Kcal/mol)	Population (%)	Conformer	No.	Energy (Kcal/mol)	Population (%)	Conformer
**3a**-1	-1081542.67031	52	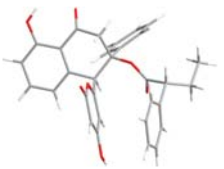	**3b**-1	-1081542.38090	54	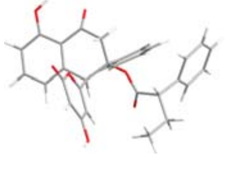
**3a**-2	-1081542.51908	40	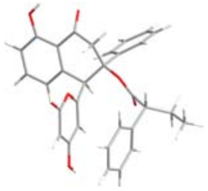	**3b**-2	-1081541.88561	23	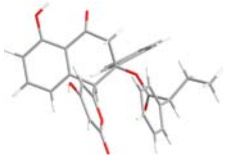
**3a**-3	-1081540.97415	3	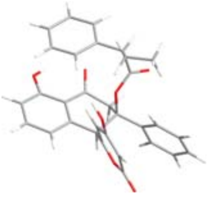	**3b**-3	-1081541.33007	9	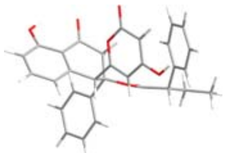
**3a**-4	-1081540.97403	3	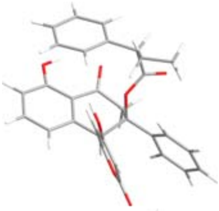	**3b**-4	-1081541.21442	7	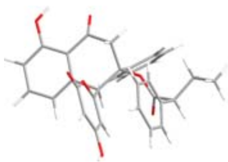
**3a**-5	-1081540.82449	2	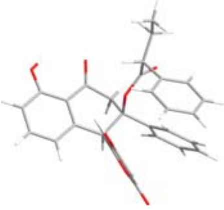	**3b**-5	-1081541.10229	6	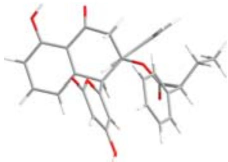

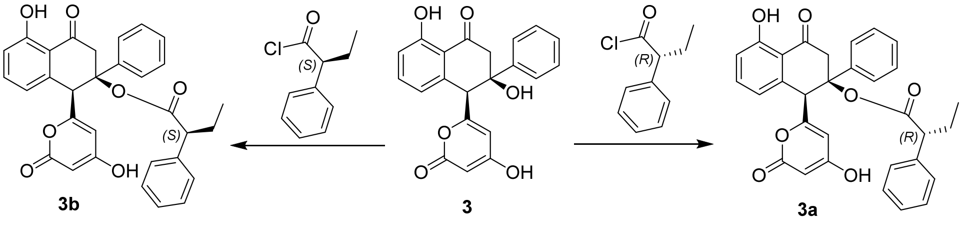

Supplement: Supplementary Information [file srep20004-s1.pdf]
